# Isolation and Genomic Characterisation of Five Novel Lytic Bacteriophages Infecting the Emerging Pathogen *Klebsiella grimontii*

**DOI:** 10.3390/v18090942

**Published:** 2026-08-28

**Authors:** Anna Tokmakova, Anna Lukianova, Mikhail Shneider, Ilia Putilov, Maria Filatova, Anna Burtseva, Konstantin M. Boyko, Yuliya Mikhailova, Andrey Shelenkov, Elizaveta Popova, Konstantin Miroshnikov

**Affiliations:** 1 Shemyakin-Ovchinnikov Institute of Bioorganic Chemistry, Russian Academy of Science, Miklukho-Maklaya Str. 16/10, 117997 Moscow, Russia; anna.zem@mail.ru (A.T.); a.al.lukianova@gmail.com (A.L.); mm_shn@mail.ru (M.S.); iaputilov@edu.hse.ru (I.P.); filat00va.maria@gmail.com (M.F.); 2 Moscow Center for Advanced Studies, Kulakova Str. 20, 123592 Moscow, Russia; a.burtseva@fbras.ru; 3 Faculty of Biology and Biotechnology, HSE University, Profsoyuznaya Str. 33, Bldg. 4, 117418 Moscow, Russia; 4 A.P. Nelyubin Institute of Pharmacy, Sechenov First Moscow State Medical University of the Ministry of Health of the Russian Federation (Sechenov University), 119048 Moscow, Russia; 5 Bach Institute of Biochemistry, Federal Research Centre “Fundamentals of Biotechnology”, Russian Academy of Sciences, Leninsky Prosp. 33, 119071 Moscow, Russia; kmb@inbi.ras.ru; 6 Central Research Institute of Epidemiology, Rospotrebnadzor, Novogireyevskaya Str. 3A, 111123 Moscow, Russia; mihailova@cmd.su (Y.M.); shelenkov@cmd.su (A.S.); 7 Faculty of Biology, Lomonosov MSU, Leninskie Gory 1, 119234 Moscow, Russia; e.a.popova1994@gmail.com

**Keywords:** *Klebsiella grimontii*, bacteriophage, phage therapy, *Przondovirus*, *Sugarlandvirus*, *Slopekvirus*, structural modelling

## Abstract

*Klebsiella grimontii* is an emerging pathogen associated with multidrug resistance. Although a single recent study reported phages against *K. grimontii*, their taxonomic diversity and structural characterisation remained limited. Here, we describe the isolation and characterisation of five novel lytic bacteriophages that specifically target *K. grimontii* strain K15g, originally isolated from a rotting iris rhizome. Phages Silvester, Lyra, Lyris, Mirion, and Helios were isolated from wastewater samples collected in the Moscow region and represent three distinct morphotypes: podovirus (Silvester), siphovirus (Lyra, Lyris, Mirion), and myovirus (Helios). Whole-genome sequencing revealed that Silvester belongs to the genus *Przondovirus* (family *Autographiviridae*); Lyra, Lyris, and Mirion are members of the genus *Sugarlandvirus* (family *Demerecviridae*); and Helios falls within the genus *Slopekvirus* (family *Straboviridae*). All five phages lack genes associated with lysogeny, virulence factors, or antibiotic resistance, indicating their suitability for therapeutic applications. Structural modelling of the receptor-binding proteins revealed diverse architectures of adsorption apparatuses, including a two-depolymerase complex in Silvester, T5-like tail fibres in the *Sugarlandvirus* phages, and a set of long and short fibres with a structured cell-puncturing device in Helios. This study presents five novel phages active against *Klebsiella grimontii* and provides a basis for further evaluation of their potential in phage therapy.

## 1. Introduction

Bacteria of the genus *Klebsiella* are well-known as opportunistic pathogens, frequently associated with multidrug resistance. Within this group, the *Klebsiella oxytoca* complex possesses blaOXY-mediated resistance and the capacity to acquire extended-spectrum β-lactamases and carbapenemases. Members of this complex cause a range of opportunistic infections in humans, including antibiotic-associated haemorrhagic colitis, urinary tract infections, and bacteraemia [1]. Although infections caused by *K. oxytoca* complex isolates are less common than those attributed to the better-studied *K. pneumoniae*, they tend to be more severe and invasive [2]. The *K. oxytoca* complex includes the recently described species *Klebsiella grimontii*. Most *K. grimontii* strains are clinical isolates derived from blood, wounds, faeces, and cases of antibiotic-associated colitis [3,4,5,6]. In a large-scale MALDI-TOF analysis, *K. grimontii* accounted for 2.4% of all typed clinical *Klebsiella* isolates, indicating its regular presence in clinical specimens rather than sporadic association with nosocomial infections [2]. *K. grimontii* has been associated with colitis, bloodstream infections, and urinary tract infections, and isolates have been found to harbour clinically relevant resistance determinants such as blaVIM-1 and mcr-9 [5,6], underscoring its potential as a reservoir of transferable resistance genes. Together, these findings establish *K. grimontii* as a newly emerging opportunistic pathogen and a reservoir of resistance genes that remains insufficiently characterised and deserves further investigation.

Phage therapy represents a promising strategy for combating clinically significant bacterial infections associated with multidrug resistance [7]. Personalised phage therapy administered in combination with antibiotics has been associated with substantial clinical improvement [8]. Successful cases of phage therapy in patients with *Klebsiella* infections have been reported [9,10]. Additionally, in vivo studies support the efficacy of this approach against pathogens of the *K. oxytoca* complex [11].

For therapeutic applications, it is critical that bacteriophages are well-characterised, strictly lytic, and lacking lysogeny-associated genes, toxins, or virulence factors [12]. Consequently, each newly characterised set of “professionally” lytic phages expands the therapeutic arsenal suitable for clinical use.

We previously isolated the *K. grimontii* strain K15g from a decaying rhizome of *Iris germanica*. Despite its environmental origin, this strain exhibited phenotypic resistance profiles typical of clinical isolates and was found to harbour blaOXY-6-4 and oqxB resistance determinants. We have also characterised the structure of its capsular polysaccharide [13], establishing K15g as a suitable model for investigating phage-mediated lysis and adsorption mechanisms.

In the present study, we describe five novel strictly lytic bacteriophages active against strain K15g. For each phage, we provide biological features, including growth and adsorption kinetics, taxonomic placement based on whole-genome sequencing, and detailed structural models of the receptor-binding proteins. Our findings expand the current knowledge of phages capable of infecting *K. grimontii* and provide a foundation for the future development of phage-based therapeutics against this pathogen.

## 2. Materials and Methods

### 2.1. Bacterial Strain

The *Klebsiella grimontii* K15g strain, isolated from a bearded iris (*Iris germanica*) plant, was used in this study. Cultivation of the bacteria was carried out using the LB-Lennox liquid medium (10 g/L of tryptone (ServiceBio, Wuhan, China), 5 g/L of yeast extract (ServiceBio), and 5 g/L of NaCl) at 37 °C. This medium, supplemented with 0.75% agar for the top layer and 1.5% agar for solid plates, was used for double-layer phage plating.

To test the pectolytic activity of K15g, crystal violet pectate (CVP) medium was used (10 g/L of sodium pectate, 0.5 g/L of KH_2_PO_4_ (pH 7.0–7.2), 0.2 g/L of MgSO_4_·7H_2_O, 15 g/L of agar, 0.01 g/L of crystal violet, and 0.33 g/L of CaCl_2_·2H_2_O (added after sterilisation).

### 2.2. Gram Staining

The Gram reaction and morphology of *K. grimontii* K15g were determined using the standard protocol of the Gram stain. Briefly, heat-fixed smears were sequentially treated with crystal violet, Gram’s iodine, acetone-alcohol decolouriser, and fuchsin. The stained preparations were then examined under oil immersion at 1000× magnification using a light microscope.

### 2.3. Phylogenetic Analysis of Klebsiella grimontii K15g

The dataset comprised 38 *Klebsiella* genome assemblies downloaded from NCBI GenBank [14]. Type strains were defined following LPSN (accessed 19 July 2026) [15]; other strains were chosen based on taxonomic annotation and assembly completeness. Coding sequences of 81 conserved proteins constituting the bacterial core genome were extracted from whole-genome assemblies of the studied strains using UBCG2 v2.0 [16]. Multiple amino acid sequence alignments were performed for each protein using MAFFT v7.505 with the L-INS-I algorithm and the BLOSUM30 matrix [17]. Aligned sequences were concatenated into a single supermatrix using AMAS v1.00, with gene boundaries specified in a partition file [18]. A maximum-likelihood phylogenetic tree was reconstructed using IQTREE v3.0.1 under a partitioned model [19]. For each partition, the best-fit amino acid substitution model was selected with ModelFinder, followed by merging partitions with identical models. As a result, the initial 81 partitions were reduced to 6 evolutionary blocks with the following models: Q.PLANT + F + R3, Q.INSECT + F + I + G4, Q.PLANT + F + I + G4, JTTDCMUT + F + I + G4, Q.INSECT + F + I, and JTT + F + G4. Tree topology was optimised using nearest-neighbour interchange (NNI) searches. Branch support was assessed via ultrafast bootstrap with 1000 pseudoreplicates. The tree was rooted on the outgroup *Escherichia coli* ATCC 11775^T^. Visualisation was performed in iTOL [20].

### 2.4. Antibiotic Sensitivity Testing

Antimicrobial susceptibility was determined using the disc diffusion method with the DIPLS-50-01 extended indicator disc set (NITsF, St. Petersburg, Russia), according to the manufacturer’s instructions. Resistance determinants were identified using ResFinder with default parameters.

### 2.5. Phage Isolation and Purification

The *Klebsiella grimontii* K15g strain was used to search for bacteriophages. Wastewater from water treatment facilities in the Moscow region (Russia) was used. The appropriate amount of dry LB medium ingredients and 5 mL of an overnight culture of K15g bacteria were added to 500 mL of each wastewater sample. The mixture was incubated overnight at 37 °C, followed by centrifugation to remove bacterial cells. The supernatant was used to isolate individual plaques by the double-layer agar method.

High-titer phage lysates were prepared from single-plaque isolates after three rounds of plaque purification. A 500 mL culture of K15g was grown to exponential phase (OD_600_ = 0.1), infected with the phage, and incubated until lysis. Chloroform (1 mL per 500 mL) was added, and cellular debris was removed by centrifugation. The cleared lysate was supplemented with PEG-8000 (10% *w*/*v*) and NaCl (0.5 M), and incubated overnight at 4 °C. The precipitate was pelleted by centrifugation (8000 *g*, 20 min, 4 °C), resuspended in SM buffer (100 mM NaCl, 8 mM MgSO_4_·7H_2_O, 50 mM Tris-HCl, pH 7.2), and further purified by centrifugation at 14,000 *g* for 2 h at 4 °C. The final pellet was resuspended in 1 mL SM buffer. Purified phage stocks were titrated by the double-layer agar method and stored at 4 °C.

### 2.6. Phage Transmission Electron Microscopy

For phage purification prior to electron microscopy, we followed the protocol described by Ackermann [21]. Briefly, 100 µL of high-titer phage lysate (approximately 10^8^ PFU/mL) was pelleted by ultracentrifugation in a fixed-angle rotor at 25,000 *g* for 1 h. The pellet was resuspended in 0.1 M ammonium acetate (pH 7.0) and re-centrifuged under the same conditions. The final sediment was used for negative staining.

Phage morphology was visualised using negative-stain transmission electron microscopy. A total of 4 μL of concentrated and purified suspension of bacteriophages was applied onto Lacey C-only 300-mesh (Ted Pella, Redding, CA, USA) grids that were glow-discharged for 60 s at 25 mA using PELCO easiGlow (TedPella, Redding, CA, USA), and subsequently stained with a 1% aqueous solution of uranyl acetate. The prepared grids were then examined using a JEM-2100 transmission electron microscope (JEOL, Tokyo, Japan) operated at an accelerating voltage of 200 kV and equipped with a Direct Electron DE-20 (Direct Electron, San Diego, CA, USA) camera.

### 2.7. Phage Adsorption and One-Step Growth Experiments

The adsorption rate was determined by infecting the exponentially growing K15g strain (OD600 = 0.3) with Silvester, Lyra, Lyris, Mirion, and Helios phages at a ratio of 1:1000 (MOI = 0.001). A 100-μL aliquot of the mixture was collected in 850 μL of SM buffer with the addition of 50 μL of chloroform immediately after infection, then collected at 1, 2, 3, 4, 5, 10, and 15 min of incubation. The resulting samples were thoroughly vortexed and then centrifuged (8000 *g*, 4 °C, 10 min) to obtain the supernatant, which was subsequently used for titration. The experiment with each phage was repeated twice.

Growth curve experiments were also performed by infecting the exponentially growing K15g strain (OD600 = 0.3) with phages. For this purpose, the exponentially growing K15g strain was centrifuged and resuspended in 0.5 mL of LB medium, then infected with the corresponding phage at a ratio of 1:100 (MOI = 0.01) and incubated for 5 min at 37 °C. Unabsorbed phage particles were removed by centrifugation (8000 *g*, 4 °C, 10 min), and the remaining pellet was resuspended in 10 mL of LB medium. Aliquots of 100 µL were collected every 5 min over two h at 37 °C and mixed with 900 µL of SM buffer. The samples were then centrifuged (8000 *g*, 4 °C, 10 min) and immediately titrated. The experiment with each phage was repeated twice. The curves show the mean values, with error bars corresponding to the standard error of the mean.

### 2.8. Phage Host Range

The host-range panel comprised the environmental strain K15g and 34 clinical *Klebsiella* isolates (mostly *K. pneumoniae* and one *K. oxytoca*) obtained from our lab collection, initially obtained directly from patients, clinics, diagnostic labs, and research institutions (Supplementary Table S1). For strains with known capsular types, whole-genome sequences were available; the remaining clinical isolates were identified by the source collections.

### 2.9. Phage Sequencing and Annotation

Phage DNAs were extracted from high-titer purified stocks following a modified CTAB protocol [22]. Library construction was performed with the Nextera DNA Library Preparation Kit (Illumina, San Diego, CA, USA), and sequencing of phage genomes was conducted on an Illumina MiSeq platform. De novo assembly of the reads into single contigs was performed using SPAdes v.3.13 [23] with default parameters. CheckV v1.0.3 [24] was used for quality control of the final phage assemblies.

Phage genomes were first annotated with Pharokka v1.9.0 [25] and subsequently verified manually using HHpred [26].

### 2.10. Phage Phylogeny and Taxonomy Studies

Pairwise intergenomic similarities for the bacteriophage genomes were determined using VIRIDIC (Virus Intergenomic Distance Calculator) [27], a tool that implements the standard algorithm endorsed by the ICTV for this purpose [28]. In a subsequent step, the phylogeny was analysed using the VICTOR (Virus Classification and Tree Building Online Resource) web server [29].

### 2.11. Structural Modelling

Structural models of phage receptor-binding proteins were generated using AlphaFold3 [30] with default parameters, and HHpred [26] was used for homology-based structure prediction and domain identification. The quality of the AlphaFold3 models was assessed using the predicted Template Modelling score (pTM) and interface predicted Template Modelling score (ipTM), which were calculated by AlphaFold3 during the modelling process. Models were visualised and analysed using ChimeraX version 1.12.

Predicted structures were validated by comparison with experimentally determined structures of related phages deposited in the Protein Data Bank (PDB), including phages T5, T4, RB43, and P1.

## 3. Results

### 3.1. Klebsiella grimontii: Isolation, Morphology, and Antibiotic Resistance

**Figure 1:**
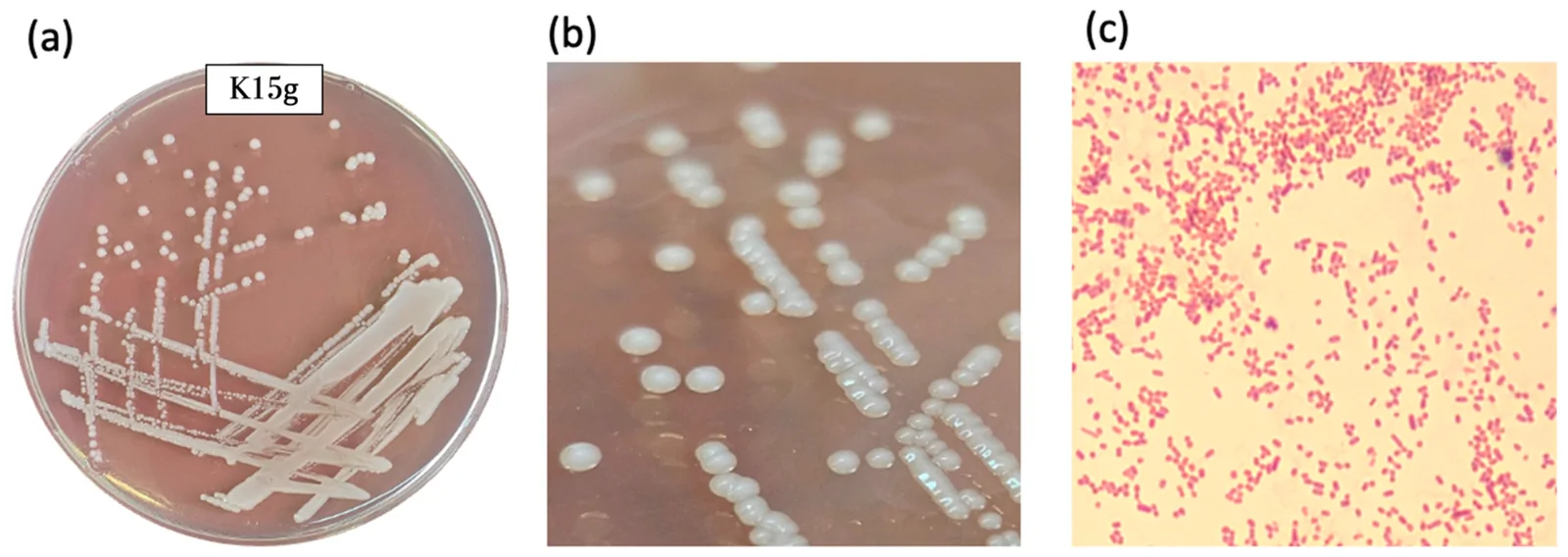
Bacterial growth of K15g on LB agar plate after 24 h incubation at 37 °C (**a**), close-up view of the colonies on the same plate ((**b**) 4× magnification), and Gram-staining of the cultured cells ((**c**) 1000× total magnification).

The strain studied in this work (*K. grimontii* K15g) was initially isolated from a rotting *Iris germanica* rhizome. Among the microbial community isolated from this source, only K15g formed pits on the CVP medium (Supplementary Figure S1), which is selective for the isolation of pectolytic strains; therefore, further close attention was paid to this isolate. With routine cultivation on LB agar, the isolate forms creamy mucoid colonies. The bacterial culture consists of small, short, round rods (Figure 1).

The draft genome (NCBI accession number: NZ_JBAMIA000000000.1) of the isolated strain was sequenced, and this confirmed the attribution of strain K15g to the *K. grimontii* species.

**Figure 2:**
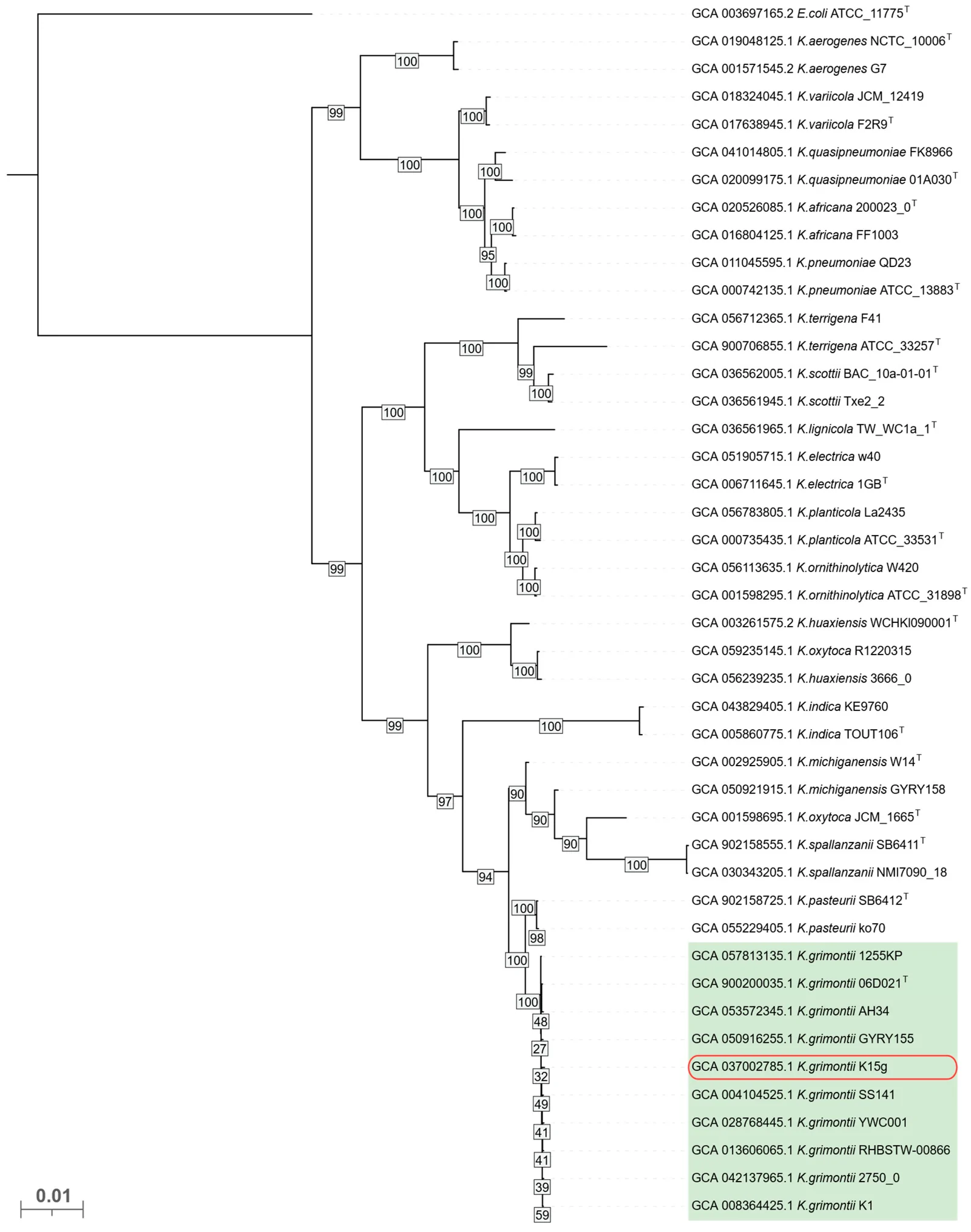
Maximum-likelihood phylogenetic tree reconstructed based on 81 conserved proteins (UBCG2) for 38 strains of the genus *Klebsiella*. The tree was rooted using *Escherichia coli* ATCC 11775^T^ as an outgroup. Branch support was assessed via ultrafast bootstrap with 1000 pseudoreplicates. Strains of *K. grimontii* are highlighted in green, and strain K15g is indicated with a red box. The scale bar represents the number of amino acid substitutions per site.

The reconstructed tree robustly resolved all major species of the genus *Klebsiella*, with high bootstrap support (≥95% for most clades). All strains annotated as *Klebsiella grimontii*, including strain K15g, formed a monophyletic clade with the type strain *K. grimontii* 06D021^T^ (bootstrap support 100%). This unambiguously confirms the assignment of strain K15g to the species *Klebsiella grimontii* (Figure 2).

Genome analysis using ResFinder revealed the presence of chromosomal beta-lactamase *bla_OXY-_*_6-4_, which was expected since the presence of bla_OXY-6_ is characteristic of the *Klebsiella grimontii* species [3]. Previously, the presence of this gene variant was stated as a feature of the phylogroup *K. oxytoca* K06, later isolated as a separate species [31]. Thus, this β-lactamase appears to be responsible for resistance to the beta-lactam antibiotics described above.

In addition, the *OqxB* gene encoding the OqxB efflux pump was detected [32]. This efflux pump is a member of the RND efflux pump family (Resistance–Nodulation–Division), which causes resistance to a wide range of antibiotics, including fluoroquinolones [33,34], which the studied strain also exhibited phenotypically. The identified resistance determinants are shown in Table 1.

**Figure 3:**
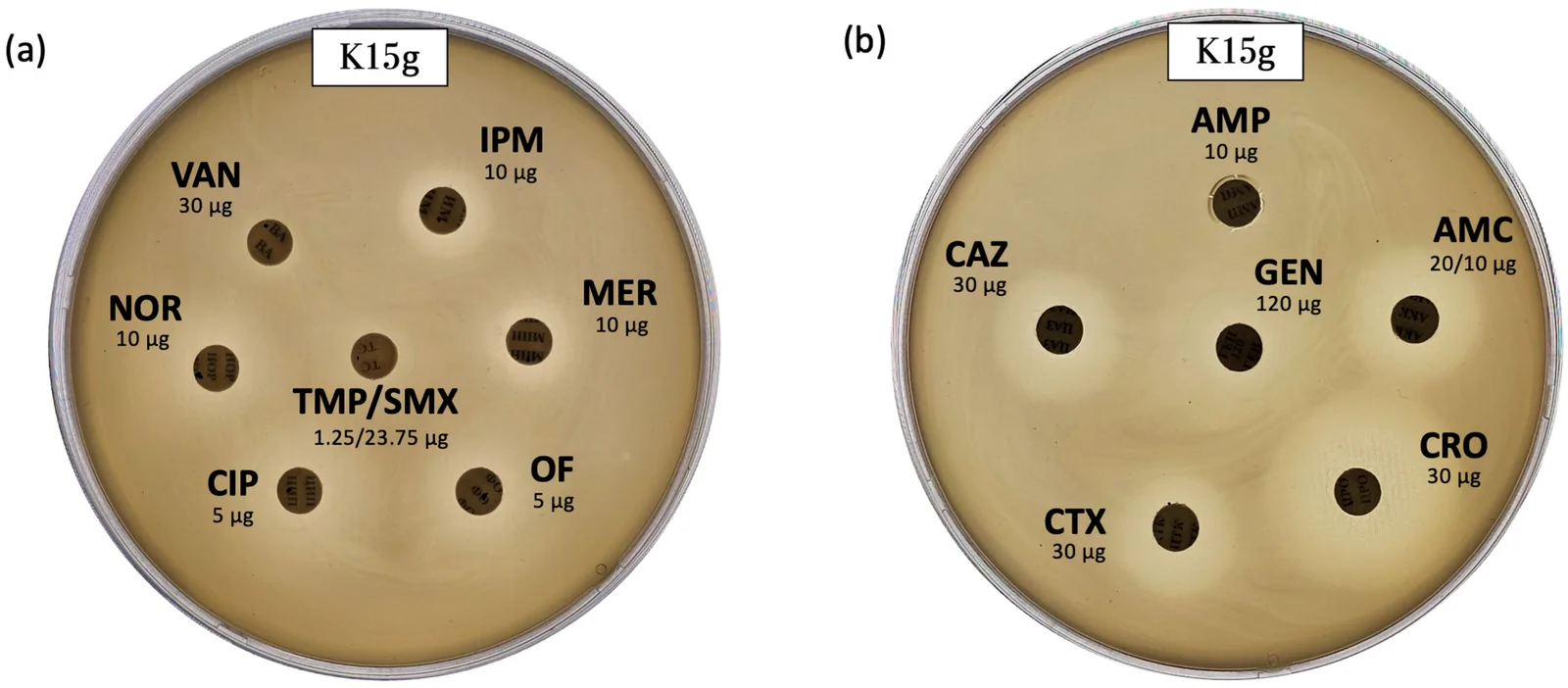
Antibiotic resistance: (**a**) IPM—imipenem (carbapenems), MER—meropenem (carbapenems), OF—ofloxacin (fluoroquinolone), Cip—ciprofloxacin (fluoroquinolone), Nor—norfloxacin (fluoroquinolone), VAN—vancomycin (glycopeptide), TMP/SMX—trimethoprim/sulfamethoxazole, (**b**) AMP—ampicillin (aminopenicillin), AMC—amoxicillin + clavulanic acid (aminopenicillin), CRO—ceftriaxone (third generation cephalosporin), CTX—cefotaxime (third generation cephalosporin), CAZ—ceftazidime (third generation cephalosporin), GEN—gentamicin (aminoglycoside).

The disc diffusion tests demonstrated resistance to ampicillin, vancomycin, and trimethoprim/sulfamethoxazole, as well as resistance to carbapenems (imipenem, meropenem) and fluoroquinolones (ofloxacin, ciprofloxacin, norfloxacin) (Figure 3). Although blaOXY-6-4 is predicted to confer resistance to several β-lactams, phenotypic testing revealed susceptibility to third-generation cephalosporins (ceftriaxone, cefotaxime, and ceftazidime) and a combination of amoxicillin with clavulanic acid and gentamicin. This difference may be due to either low expression of the resistance determinant under the conditions tested or additional regulatory mechanisms [35].

### 3.2. Bacteriophages Active Against K. grimontii K15g

#### 3.2.1. Isolation and Morphology

Due to the antibiotic resistance demonstrated by the isolate, the search for lytic bacteriophages exhibiting activity against *K. grimontii* is of considerable scientific and practical significance. Consequently, wastewater samples were collected from various treatment facilities near Moscow for phage isolation (Table 2). Five distinct bacteriophages were isolated, subjected to detailed examination, and their whole genomes were sequenced.

**Figure 4:**
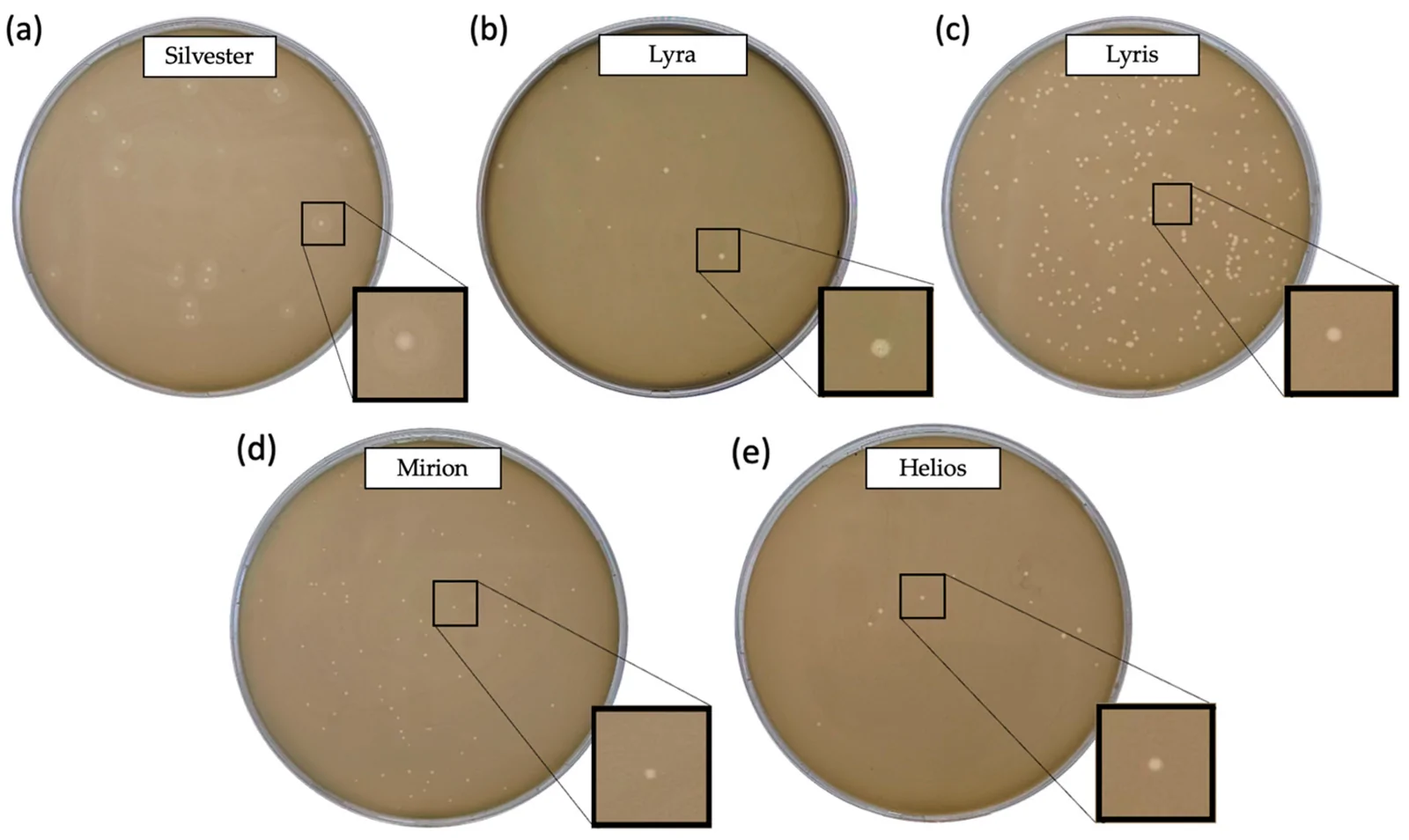
Plaque pattern formed on 0.75% top agar by *Klebsiella* phages: (**a**) phage Silvester, (**b**) phage Lyra, (**c**) phage Lyris, (**d**) phage Mirion, and (**e**) phage Helios. The insets show a 4× magnified view of single plaques.

Among the isolated phages, most of them produced medium-sized, circular plaques with well-defined edges and were without a surrounding halo on 0.75% top agar (Table 2 and Figure 4). Bacteriophage Silvester was the exception to this observation due to its translucent halo around the plaques. It is noteworthy that such morphological characteristics typically suggest the presence in the bacteriophage genome of a gene encoding an enzyme that leads to the degradation, specifically the depolymerisation, of the bacterial external polysaccharide.

**Figure 5:**
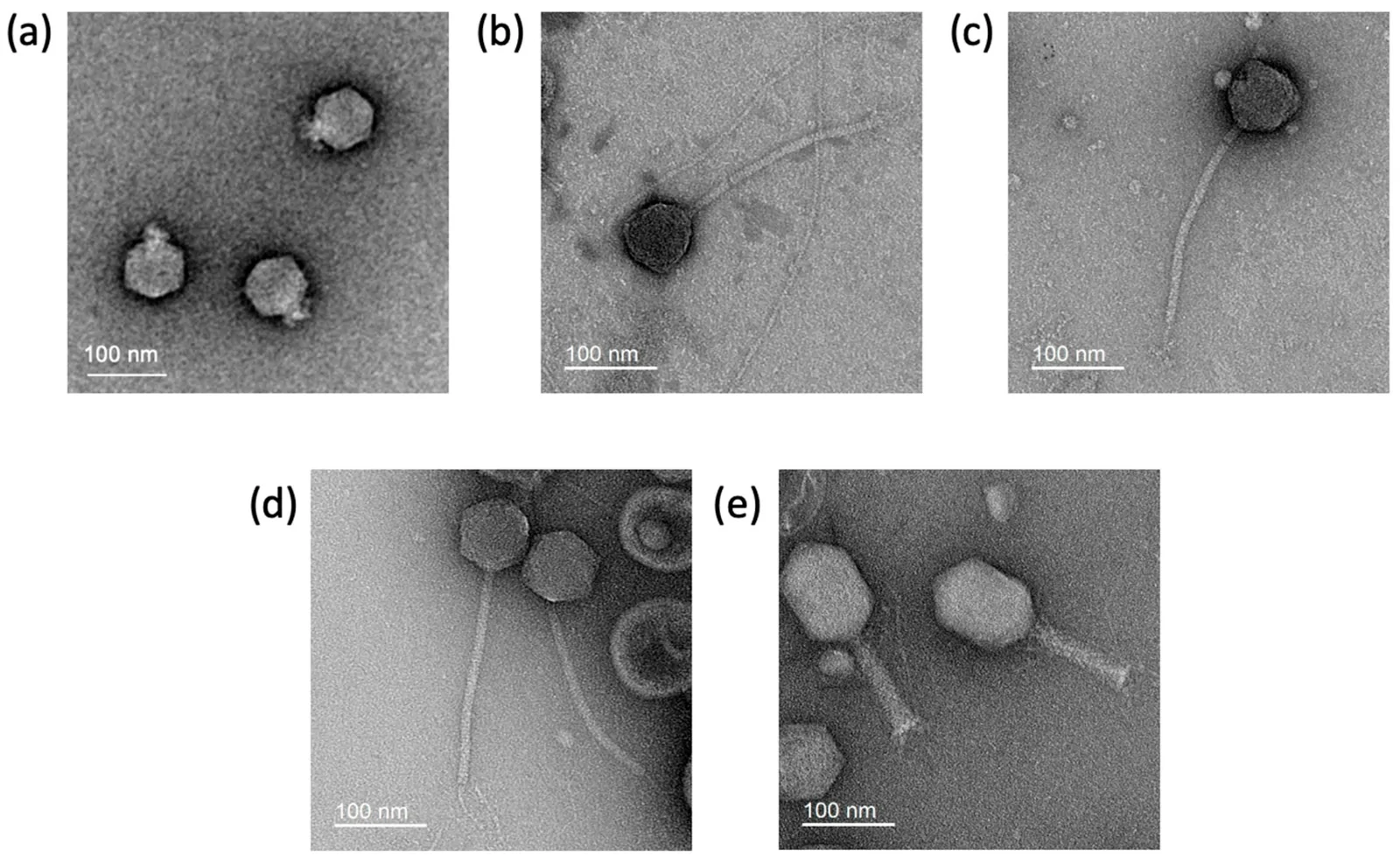
Ultrastructure of isolated bacteriophages under transmission electron microscopy: (**a**) phage Silvester, (**b**) phage Lyra, (**c**) phage Lyris, (**d**) phage Mirion, and (**e**) phage Helios.

The examination of the morphology of the isolated bacteriophages via transmission electron microscopy (TEM) revealed that bacteriophages Lyra, Lyris, and Mirion exhibit a similar siphovirus morphology, characterised by a long non-contractile tail measuring 259 ± 12 nm in length and a capsid with a diameter of 74.6 ± 5 nm. In contrast, bacteriophage Helios displayed a morphology typical of myoviruses that feature a contractile tail of 118 ± 11 nm and a capsid measuring 109 ± 6 nm in diameter. The sole bacteriophage exhibiting a halo, Silvester, was identified as a podovirus, characterised by a short tail and a capsid measuring approximately 75 nm in diameter (Figure 5).

#### 3.2.2. Adsorption and One-Step Growth

**Figure 6:**
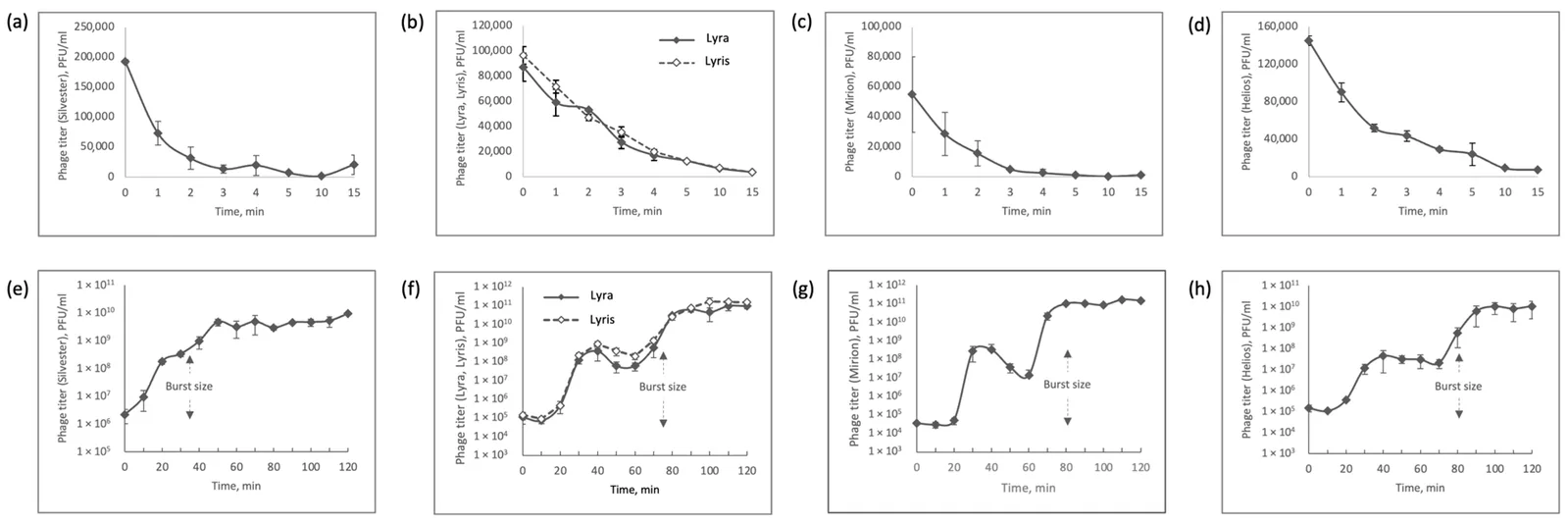
Adsorption curves (top row) and single-step growth curves (bottom row) of bacteriophages: (**a**) adsorption curve of phage Silvester, (**b**) adsorption curves of phages Lyra and Lyris, (**c**) adsorption curve of phage Mirion, (**d**) adsorption curve of phage Helios, (**e**) growth curve of phage Silvester, (**f**) growth curves of phages Lyra and Lyris, (**g**) growth curve of phage Mirion, and (**h**) growth curve of phage Helios. All graphs plot the mean concentrations of free phage particles at each time point, with error bars denoting the standard error of the mean. The fitted adsorption curves are shown in Supplementary Figure S2.

All siphovirid phages demonstrated similar one-step growth dynamics (Figure 6). Adsorption to host cells was fast in all cases; however, the adsorption rate constants, determined from linear regression of the adsorption curves, were higher for Lyra and Lyris (4.972 × 10^−10^ mL/min, R^2^ = 0.96) than for Mirion (1.088 × 10^−10^ mL/min, R^2^ = 0.99). A latent period of approximately 20 min was followed by a burst that reached a titre of 147 PFU/cell for phage Mirion, 123 PFU/cell for phage Lyra, and 337 PFU/cell for phage Lyris. This was followed by a brief plateau accompanied by a slight decrease in titre. The plotted graphs for bacteriophages Lyra and Lyris were nearly identical, demonstrating a latent period of 15 min, a burst duration of 25 min, and a second burst occurring after an additional 20 min. In the case of phage Mirion, the latent period was 20 min, the burst time was 20 min, and the second plateau lasted 45 min.

For the myoviral bacteriophage Helios, similar growth dynamics were characterised by a short latent period of 20 min and two separate bursts during the observation period. The burst size was 11 PFU/cell. The adsorption rate constant was determined as 1.002 × 10^−9^ mL/min, R^2^ = 0.92.

In contrast, the podophage Silvester exhibited a marked difference, presenting a short latent period and achieving a plateau following a burst, amounting to 103 PFU/cell. This phage displayed the highest adsorption rate among those tested at 1.382 × 10^−9^ mL/min, R^2^ = 0.98.

Host-range testing (Supplementary Table S1) showed that all five phages were specific to *K. grimontii* and did not infect any of the tested *K. pneumoniae* or *K. oxytoca* strains. In addition to the environmental strain *K. grimontii* K15g, all phages except Silvester lysed isolate *K. grimontii* 1621, while Lyra, Lyris, and Mirion also lysed isolate *K. grimontii* 3770. The remaining clinical *K. grimontii* isolates were resistant to all phages.

### 3.3. Genomic and Taxonomic Analyses of Klebsiella grimontii Phages

#### 3.3.1. General Genomic Features

Silvester has the smallest genome, with 49 predicted genes and a GC content of 52.2% (Supplementary Figure S3). It contains no tRNA genes. The genome is available under accession number PV506378.

Genomes of phages Lyra, Lyris, and Mirion (accession numbers PZ305518, PZ319654.1, and PZ319655) (Supplementary Figure S4) had minimal differences and shared high similarity with each other along their entire length (Supplementary Figure S5). They ranged from 111,013 to 112,672 bp, with GC contents of 45.4–45.6% and 178–186 predicted proteins, including 23–24 tRNAs. Helios (accession number PZ273796) has the largest genome (174,645 bp) and encodes 285 proteins, with a single tRNA gene. Its GC content is 41.9% (Supplementary Figure S6).

The genome integration/exclusion module necessary for the lysogenic life cycle, as well as virulence factors or antibiotic resistance genes, was not found in any of the five phages.

#### 3.3.2. Phylogeny and Taxonomy

**Figure 7:**
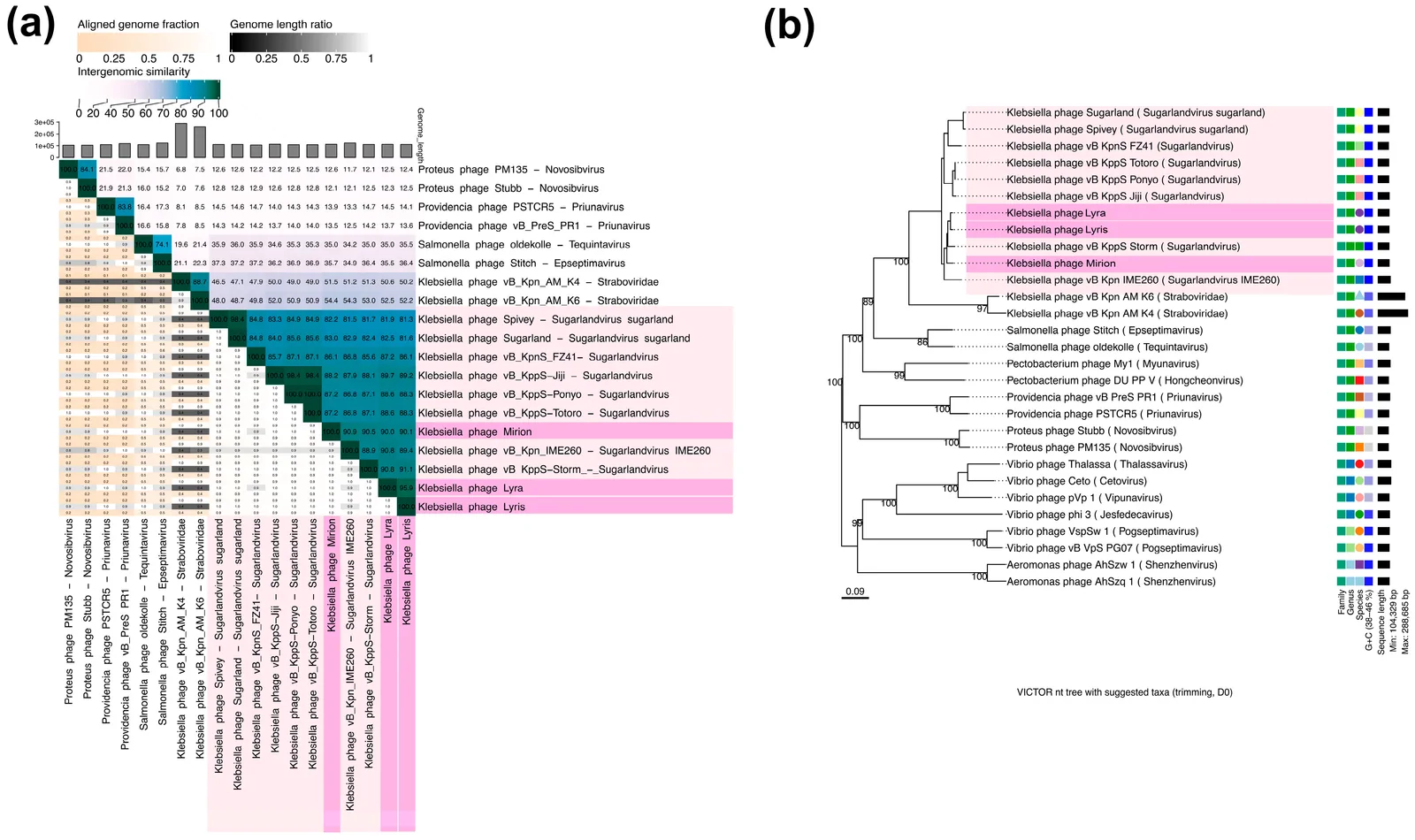
(**a**) VIRIDIC matrix based on the average nucleotide identity of *Klebsiella* phages Lyris, Lyra, Mirion, and other members of the genus *Sugarlandvirus*, as well as members of subfamily *Slopekvirinae*. The values in the cells correspond to calculated ANI values. The colour coding corresponds to the values in the legend above. (**b**) Phylogenetic tree generated with the Genome-BLAST Distance Phylogeny (GBDP) method, using the D0 formula, showing the relationship of *Klebsiella* phages Lyris, Lyra, and Mirion with representatives of *Sugarlandvirus* and *Slopekvirinae*. The numbers above the branches are GBDP pseudo-bootstrap support values from 100 replications. The branch lengths of the resulting VICTOR trees are scaled in terms of the respective distance formula used.

In the NCBI BLAST 2.17.0 searches, bacteriophages Lyris, Lyra, and Mirion have the highest degree of similarity to known bacteriophages of the genus *Sugarlandvirus* (Family *Demerecviridae*). According to the average nucleotide identity values obtained using VIRIDIC, Lyris and Lyra appear to be members of the same species within the *Sugarlandvirus* genus, as they share above 95% ANI with each other and cluster only with each other (Figure 7a). Phylogenetic data obtained using VICTOR grouped the isolated bacteriophages into a clade with other *Sugarlandviruses* and also suggest that Lyris and Lyra belong to one species and Mirion to another (Figure 7b).

**Figure 8:**
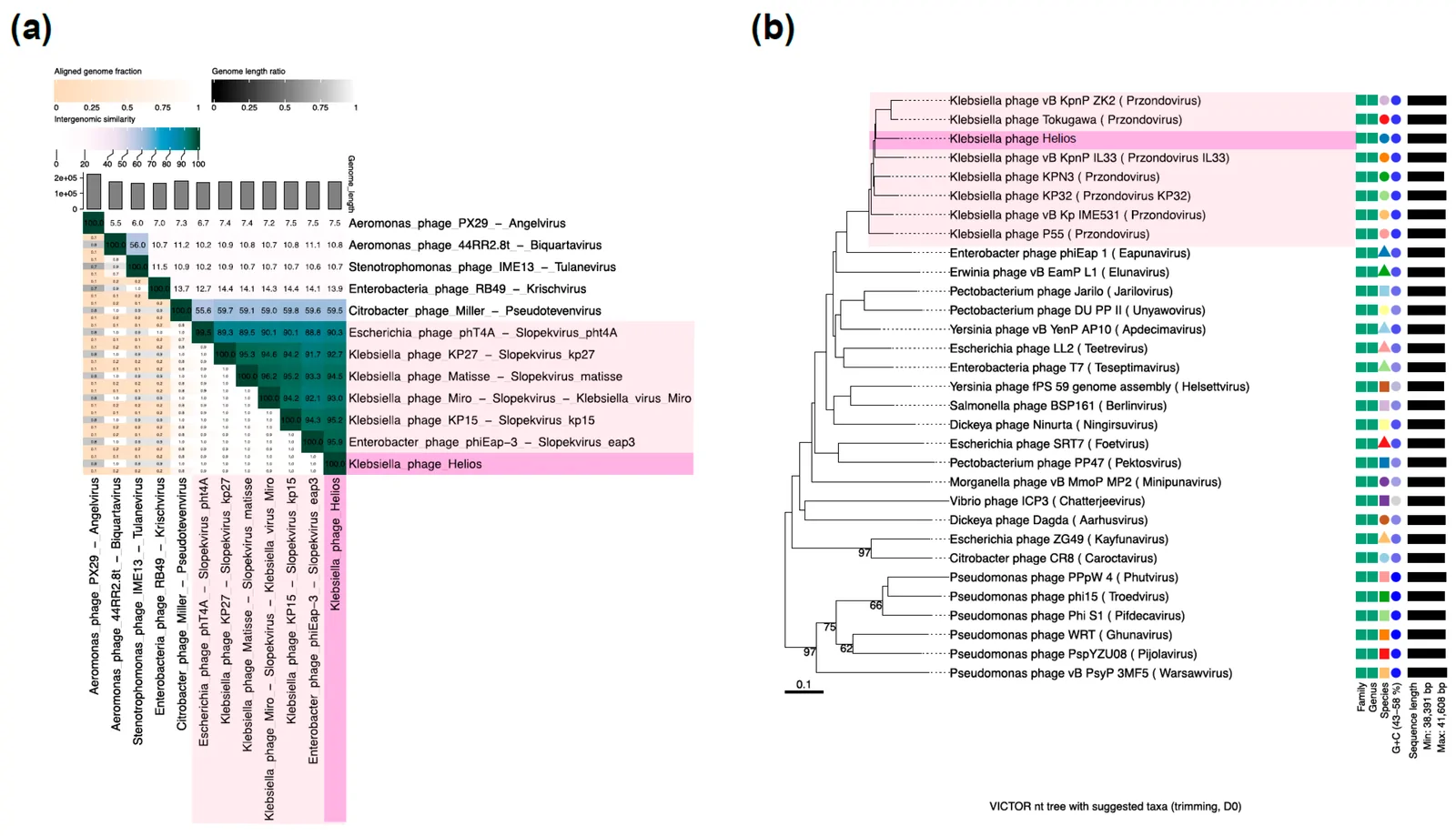
(**a**) VIRIDIC matrix based on the average nucleotide identity of *Klebsiella* phage Helios and other members of the genus *Slopekvirus*, as well as members of subfamily *Slopekvirinae*. The values in the cells correspond to calculated ANI values. The colour coding corresponds to the values in the legend above. (**b**) Phylogenetic tree generated with the Genome-BLAST Distance Phylogeny (GBDP) method, using the D0 formula, showing the relationship of *Klebsiella* phage Helios with representatives of *Slopekvirus* and *Slopekvirinae*. The numbers above the branches are GBDP pseudo-bootstrap support values from 100 replications. The branch lengths of the resulting VICTOR trees are scaled in terms of the respective distance formula used.

The genome of the bacteriophage Helios, when searched in NCBI BLAST, shows the greatest similarity to the genomes of previously published *Slopekvirus* bacteriophages (Family *Straboviridae*). The VICTOR phylogeny groups the bacteriophage in a common clade with other *Slopekviruses*, and according to the ANI values obtained using VIRIDIC, the bacteriophage we isolated shares high similarity, above 95%, with a number of previously published genomes (Figure 8).

**Figure 9:**
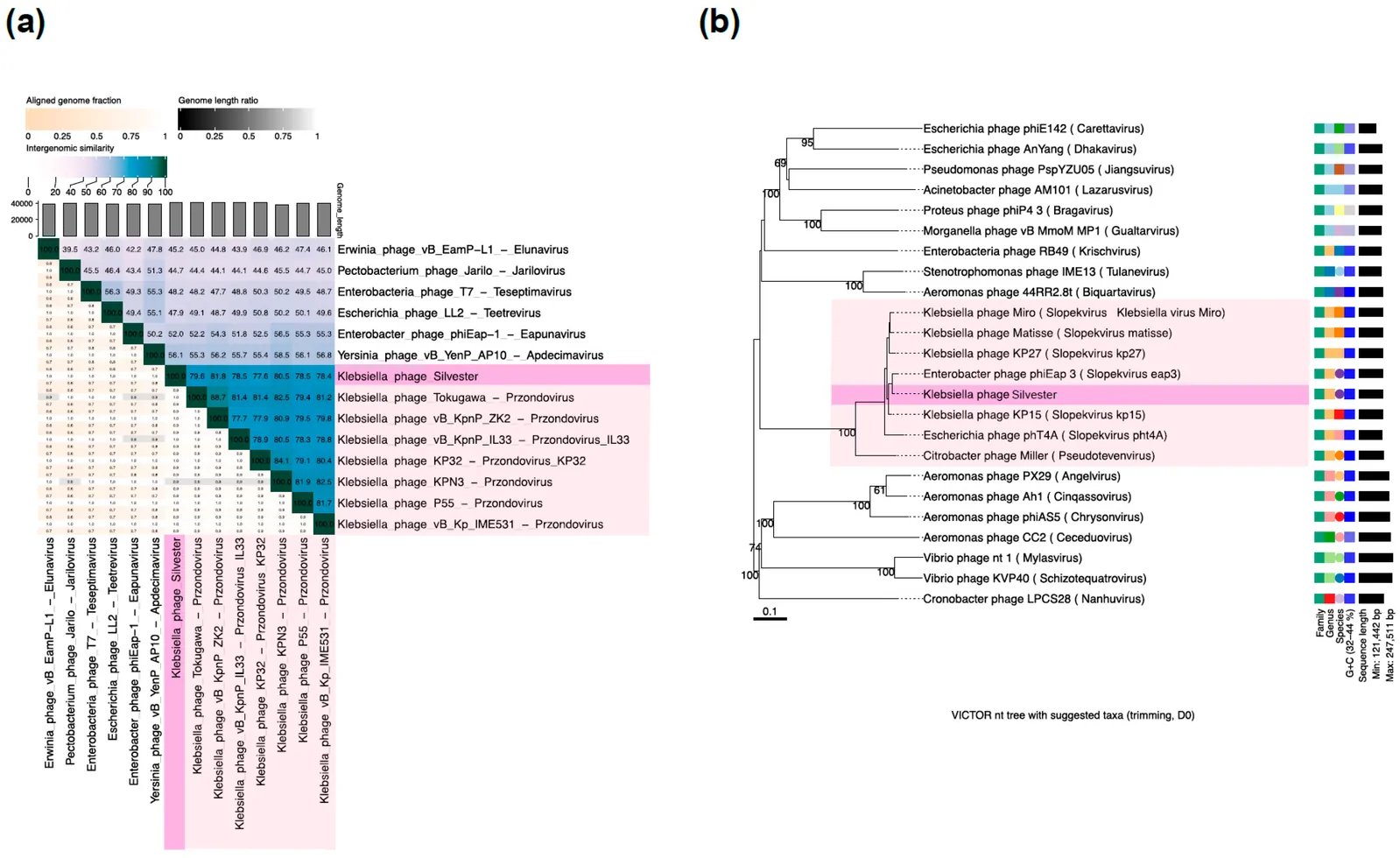
(**a**) VIRIDIC matrix based on the average nucleotide identity of *Klebsiella* phage Silvester and other members of the genus *Przondovirus*, as well as members of subfamily *Studiervirinae*. The values in the cells correspond to calculated ANI values. The colour coding corresponds to the values in the legend above. (**b**) Phylogenetic tree generated with the Genome-BLAST Distance Phylogeny (GBDP) method, using the D0 formula, showing the relationship of *Klebsiella* phage Silvester with other representatives of *Przondovirus* and *Studiervirinae*. The numbers above branches are GBDP pseudo-bootstrap support values from 100 replications. The branch lengths of the resulting VICTOR trees are scaled in terms of the respective distance formula used.

Bacteriophage Silvester, as a result of a similar analysis, was determined to belong to the genus *Przondovirus* of the family *Autographiviridae* (Figure 9).

#### 3.3.3. Adsorption Apparatus

Understanding the interaction between phage and host cells is impossible without knowing the structure of the phage adsorption apparatus. The accumulated data on the structure of phage particles, obtained through cryoelectronic reconstruction and protein structure modelling methods, allow us to examine in detail the adsorption apparatuses and structures of the receptor-binding proteins (RBPs) of the phages described in this article.

##### Phage Silvester Receptor-Binding Proteins (RBPs)

**Figure 10:**
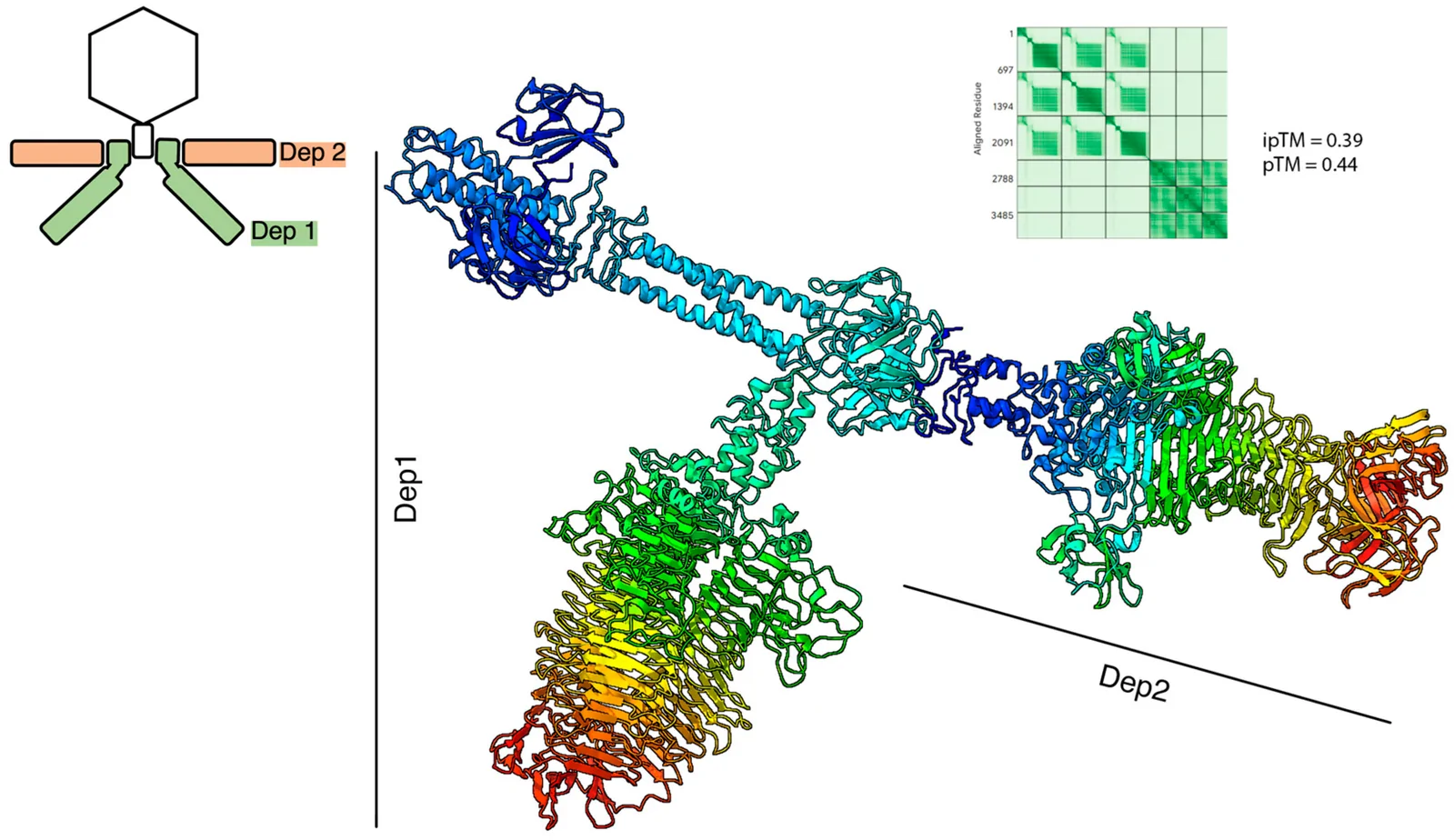
Model of the tail spikes of phage Silvester.

The phage Silvester particle is equipped with a complex of two depolymerases. Modelling of the depolymerase complex showed that gp42 depolymerase I (NCBI ID YDD23258) attaches to the tail of the phage particle in the same way as the phage T7 fibre [36]. The trimer of tail spike I has an asymmetric structure (Figure 10). A side β-structural knob, located in the middle of the oligomer molecule, is similar to the structure revealed as the binding domain XD of the tail spikes of phage CBA120 [37,38]. N-terminus of tail spike II (NCBI ID: YDD23259) attaches to this knob structure (Figure 10). Earlier, we obtained a model with the same structure for the tail spike complex of phage Kiwi [39], also belonging to the genus *Przondovirus*. We experimentally established [13] that depolymerase I specifically recognises and cleaves the CPS of strain K15g. However, we were unable to identify the substrate of depolymerase II among the strains of our collection of *Klebsiella* sp. According to the structural organisation of the molecule, depolymerase I can be assigned to class IE (depolymerase with a short C-terminal domain), and depolymerase II belongs to class 2A (depolymerase with an insertional domain) [40].

##### Tail Fibres of Phages Lyra, Lyris, and Mirion

Phages Lyra, Lyris, and Mirion belong to the genus *Sugarlandvirus*, which, in turn, belongs to the family *Demerecviridae* [41], just like the genus *Tequintavirus*. The structures of particles and receptor apparatuses of phages T5 and DT57, belonging to the genus *Tequintavirus*, were determined by cryo-electron microscopy [42,43]. The bioinformatic analysis of predicted structural proteomes of phages Lyra, Lyris, and Mirion enables us to assume substantial similarity of the structure to phage T5 (Table 3).

**Figure 11:**
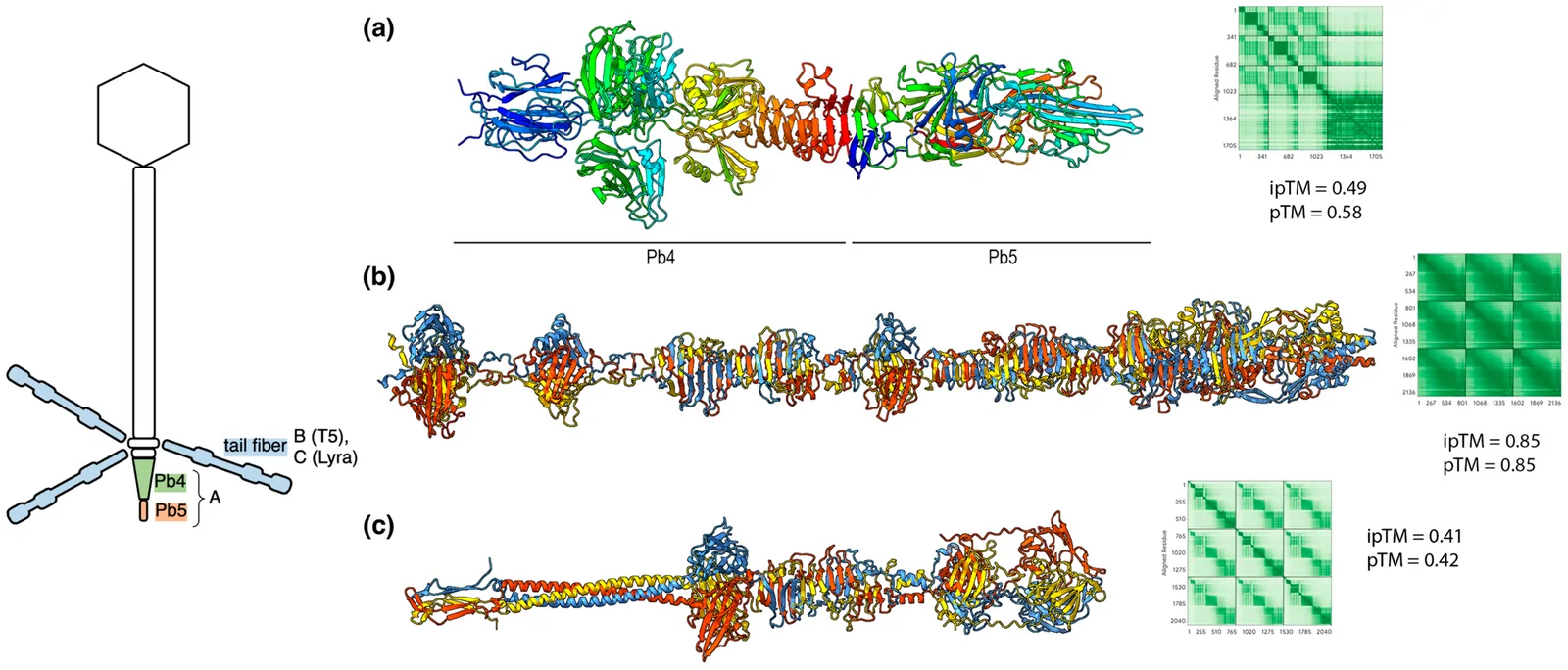
(**a**) Model of the C-terminal part of the trimer of Pb4 protein (Gly2628–Arg3446) and Pb5 protein complex produced by AlphaFold3, phage Lyra. (**b**) Model of the trimer of L-shaped tail fibre protein (Met210—Met1396) produced by AlphaFold3, phage T5; the C-terminal chaperone is not processed. (**c**) Model of the trimer of tail fibre protein produced by AlphaFold3, phage Lyra.

While the overall protein composition of the phages under consideration is similar to that of phage T5, some structural components, such as the short tail fibres (STF), Pb4, and Pb5 proteins, have species-specific features. As the phages are very similar, we use phage Lyra as an example for structural comparisons. A sequence comparison of the Pb4 and Pb5 orthologues among Lyra, Lyris, and Mirion revealed high similarity, with 90–97% and 81–100% amino acid identity, respectively, indicating that their overall structures are likely conserved. Accordingly, we used Lyra as a representative for structural modelling. The orthologs of the T5 pb4 protein have a large size of 3446–3454aa (compared to 688aa for phage T5). Modelling by HHpred and AlphaFold3 demonstrates that the structure of the trimer of this protein is an extended fibre comprising b-structural domains (lectin-fold-like and Ig-fold-like) in variable combinations, joined by triple collagen helix segments. Suggestedly, the b-structural domains perform carbohydrate-binding, and the collagen inserts provide flexibility to the overall structure. The C-terminal domain of Pb4 (residues Gly2628–Arg3446) is modelled as a triple b-prism forming a platform where the b-structural monomer of Pb5, responsible for binding to the receptor on the cell surface, is attached (Figure 11a). Structurally, the Pb4-Pb5 complex of phage Lyra is very similar to its analogue from phage T5 [42]. It can be assumed that the target of Pb5 is a protein on the cell outer membrane, as has been shown for several other phages of the family *Demerecviridae* [44].

A comparison of the AlphaFold3 structural models of the trimers of short tail fibres of phage Lyra and the L-shaped tail fibre of phage T5 demonstrates a similarity based on a combination of common structural elements, such as pyocin knobs [45] and LTF proximal subunits [46] (Figure 11b,c). This property of combining elements within the structure of various bacteriophage fibres has been previously described in detail [47]. However, the receptor domains of the fibres of phage T5 [48] and phage Lyra are completely different.

##### Tail Fibres of Phage Helios

**Figure 12:**
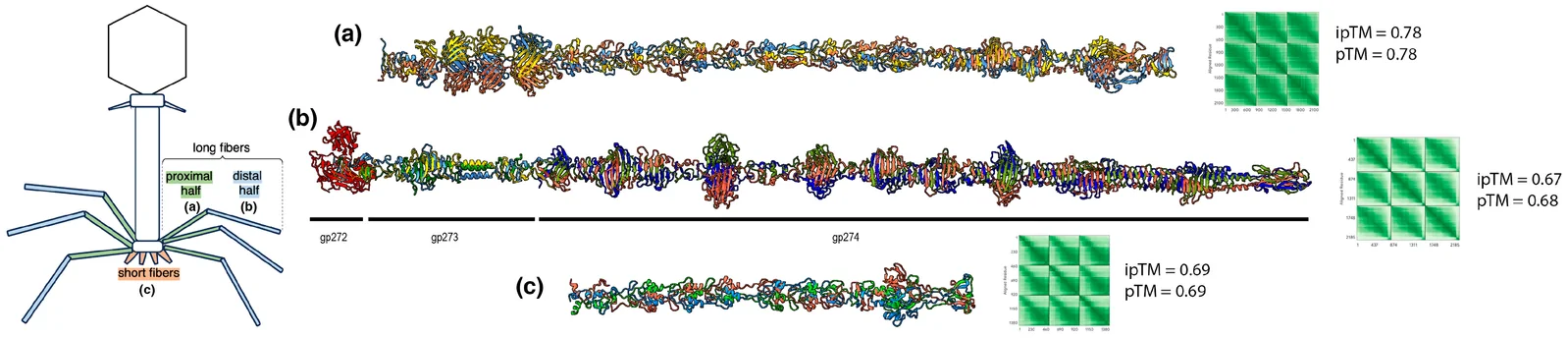
Fibres of phage Helios. (**a**) Model of the trimer of the proximal half of long tail fibres (gp271) generated by the AlphaFold3 programme. (**b**) Model of the complex of the distal half of long tail fibres generated by the AlphaFold3 programme (gp272, gp273, and gp274). (**c**) Model of the trimer of the short tail fibres gp210 (NCBI ID: YDX46262).

We examined the features of phage Helios infection apparatus compared to other *Straboviridae* phages. Similar to other phages of this family, the RBP of phage Helios includes a set of long (LTF) and short fibres (Figure 12). The long fibres consist of a proximal part, a trimer of gp271 (NCBI ID: YDX46323), and a distal part composed of a single copy of gp272 (NCBI ID: YDX46324), forming the LTF knee [49] and three copies of proteins gp273 (NCBI ID: YDX46325) and gp274 (NCBI ID: YDX46326).

**Figure 13:**
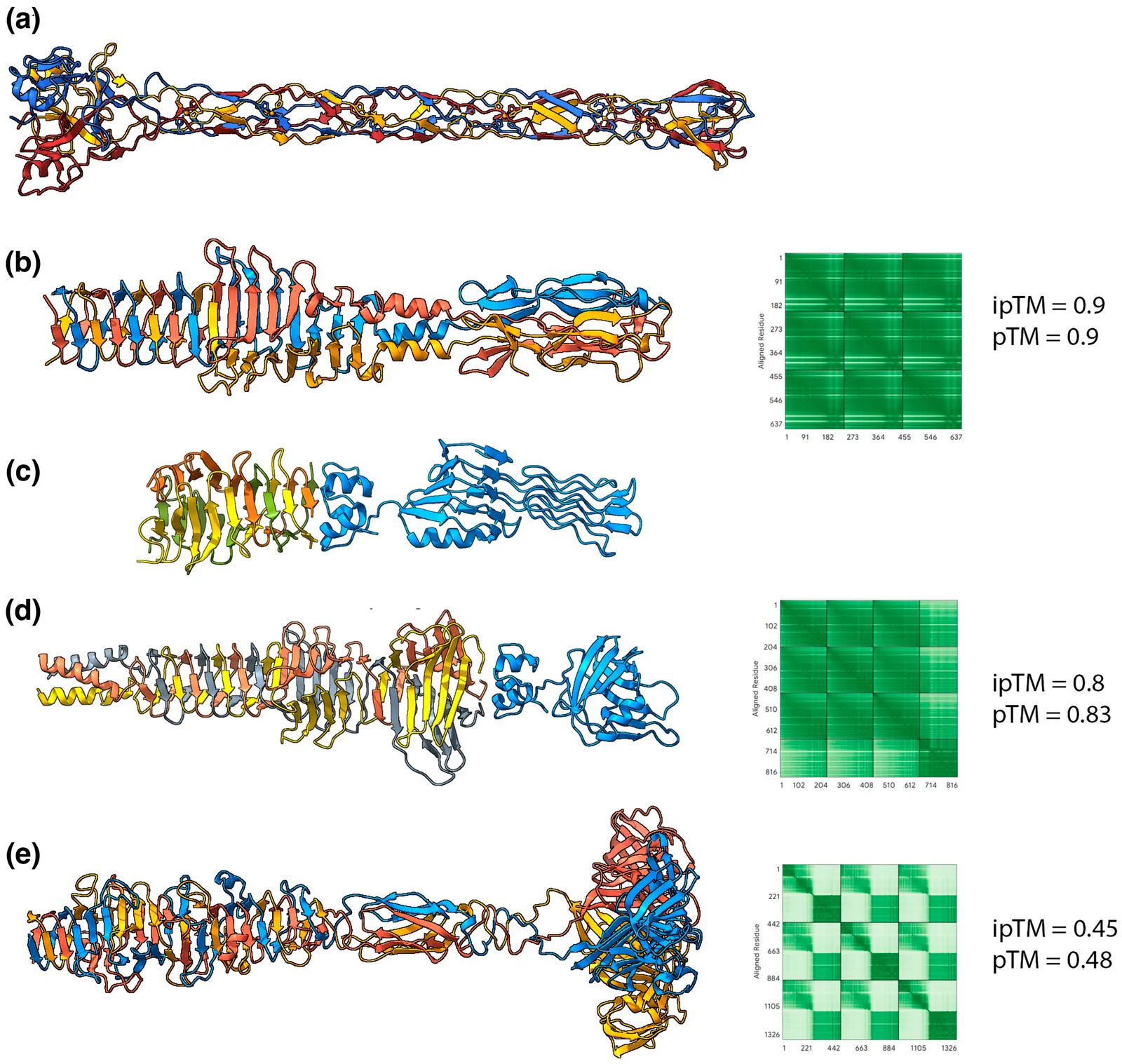
Variability of receptor domains of the long tail fibres of phages belonging to family *Straboviridae*. (**a**) Atomic structure of the C-terminal part of gp37 of phage T4 (PDB ID: 2XGF) [52,54]. (**b**) Model of the C-terminal trimeric part of gp274 (Thr1025–Thr1237) of phage Helios. (**c**) Atomic structure of the C-terminal parts of gp37 and gp38 (blue) of phage S16 (PDB ID: 6F45) [51]. (**d**) Model of the C-terminal part of the trimeric distal part of LTF gp266 (NCBI ID: YP_009607461) (Thr958—Val1171) and tail fibre adhesin gp267 (blue) (NCBI ID: YP_009607462) of *Klebsiella* phage Miro (NCBI ID: NC_041981) generated by AlphaFold3. (**e**) Model of the C-terminal part of the trimeric distal part of LTF gp149 (NCBI ID: YP_009887012) (Pro807—Val1248) of *Acinetobacter* phage Lazarus (NCBI ID: NC_049493) generated by AlphaFold3.

This composition of LTF was first described in phage T4 (gp34, gp35, gp36, and gp37), and this structural organisation is typical for all phages of the *Straboviridae* family. At the same time, the receptor domains of long fibres show a significant diversity of structures. Here, we tried to summarise the diversity of the structural variants of the LTF receptor-binding domains of the different *Straboviridae* phages (Figure 13). As a rule, LTF utilises outer membrane proteins as a receptor, but sometimes it is able to use an outer carbohydrate moiety [44,50,51]. In some phages, receptor binding is delegated to a special protein, gp38, which is located at the tip of gp37. While the structure of gp38 of phage S16 was established by X-ray crystallography [51] (Figure 13c), another variant of gp38 was modelled by AlphaFold3 for *Klebsiella* phage Miro (NCBI ID: NC_041981) (Figure 13d). The OmpC-recognising tip of gp37 of phage T4 has a needle-like structure [52] (Figure 13a). It was recently shown that *Straboviridae* phages infecting *A. baumannii* use the receptor porin CarO [53]. The modelled distal tip of gp149 (gp37 ortholog) of *Acinetobacter* phage Lazarus demonstrates an original flat triangular structure (Figure 13e).

**Figure 14:**
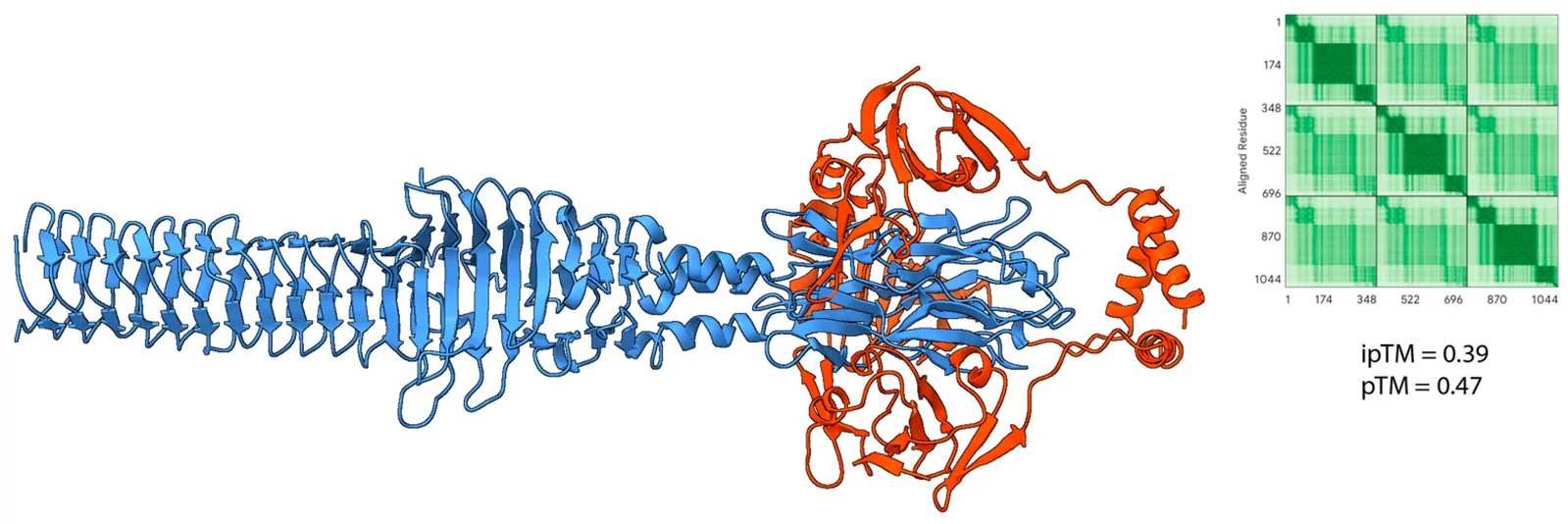
Model of the C-terminal part of trimeric gp274 protein (blue) (Asp1005—Thr1237) and chaperone gp275 (orange) complex produced by AlphaFold3, phage Helios.

The predicted structure of the receptor domain of LTF of phage Helios (Figure 14, blue) is similar to previously described ones of phage Mu fibre Tfib_Mu_ (PDB ID: 5YVQ) [46] and *Vequintavirinae* phage MaxBurger [47]. The protein gp275 (NCBI ID: YDX46327) was recognised as an LTF chaperone, which forms a complex with the distal LTF subunit in a manner similar to Tfib_Mu_:Tfa_Mu_ [46] (Figure 14, orange).

**Figure 15:**
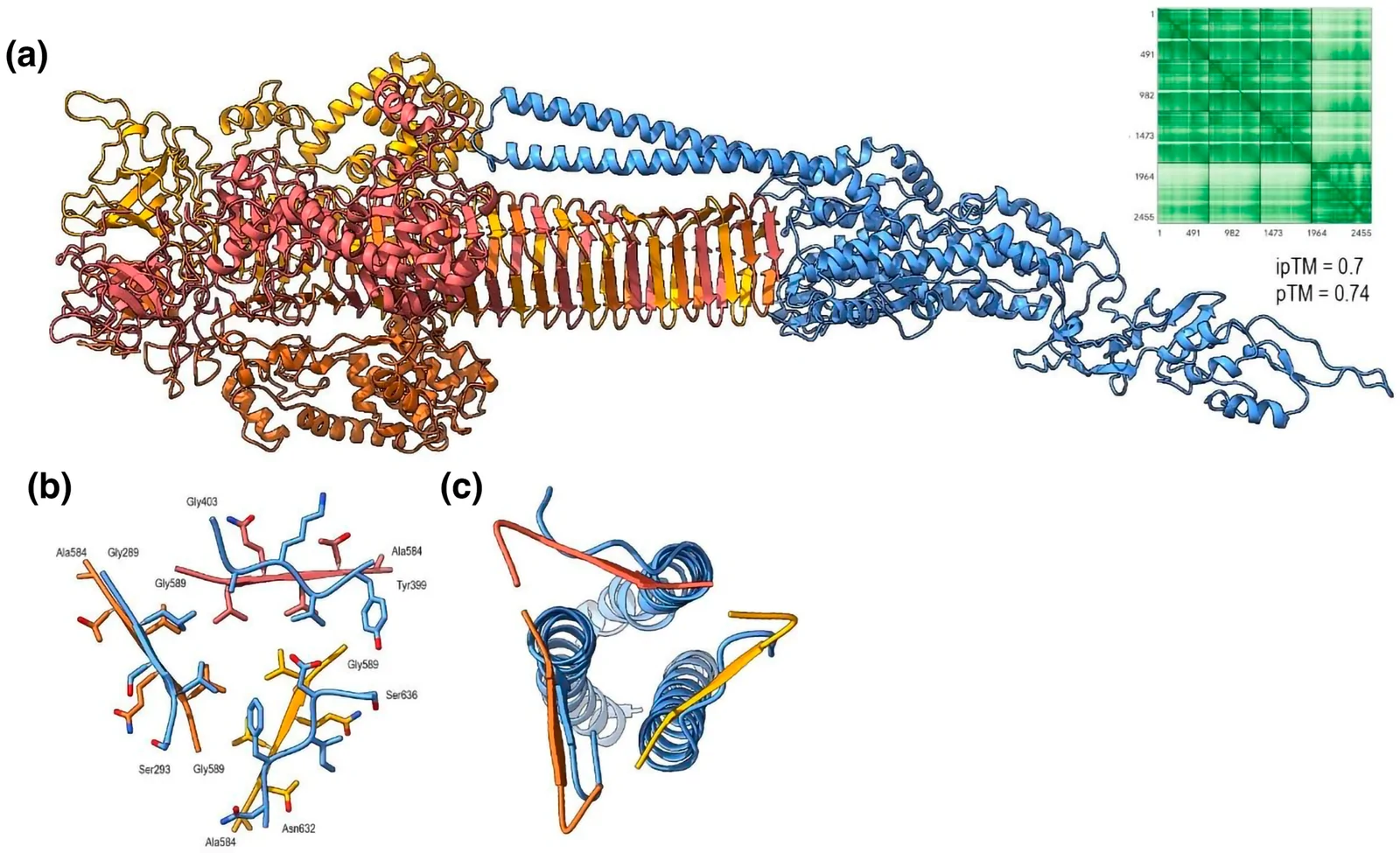
(**a**) Model of the trimer of phage Helios cell-puncturing devices gp202 (yellow and brown) and gp203 (blue) generated by AlphaFold3. (**b**) Model of the intermolecular interaction interface of the gp202 and gp203 trimer. Amino acid residues of the last β-strand of the gp202 prism (Ala584—Gly589) and the gp203 parts interacting with them are shown. (**c**) Model of the triple coiled-coil core of the gp203 structure (Phe287—Ile327, Glu398—Trp440, Lys631—Ile670). The last β-strand of the gp202 trimer prism (Gly583—Gly589) is shown.

Contractile systems use puncturing devices to penetrate outer cell membranes. Phages of the *Straboviridae* family possess a genetic module consisting of tail lysozyme gene 5 and two small genes 5.1 and 5.4. Phage T4 protein 5.4 (PAAR protein) forms a sharp pyramid on the tip of the gp5 trimer [55,56]. Genomic analysis revealed that some phages of the *Straboviridae* family lack genes 5.1 and 5.4, but the corresponding genomic position is occupied by a gene encoding a large protein whose functions have not been established experimentally. It was suggested that the function of this protein is to create a sharp tip on gp5, as gp5.4 does [56]. We identified gp203 (NCBI ID: YDX46255) in the genome of phage Helios as such a protein. The model of the gp202 trimer (NCBI ID: YDX46254, gp5 of phage T4 homologue) and gp203 produced by the AlphaFold3 programme (Figure 15a) is similar to that obtained for phage RB43 [56].

The model of the interaction between the gp202 trimer and gp203 demonstrated similarity to the gp5/gp5.4 contact. The triangular tip of gp202 forms a hydrophobic interaction with the gp203 base in the core region, and polar amino acids interact on the outside (Figure 15b). Within the gp203 structure core, the model demonstrates a triple coiled-coil (Figure 15c) domain, which may impart the high degree of rigidity necessary to penetrate the plasma membrane. Another notable feature of this structure is the loop at the tip, which provides an initial interaction with the membrane when penetration occurs.

**Figure 16:**
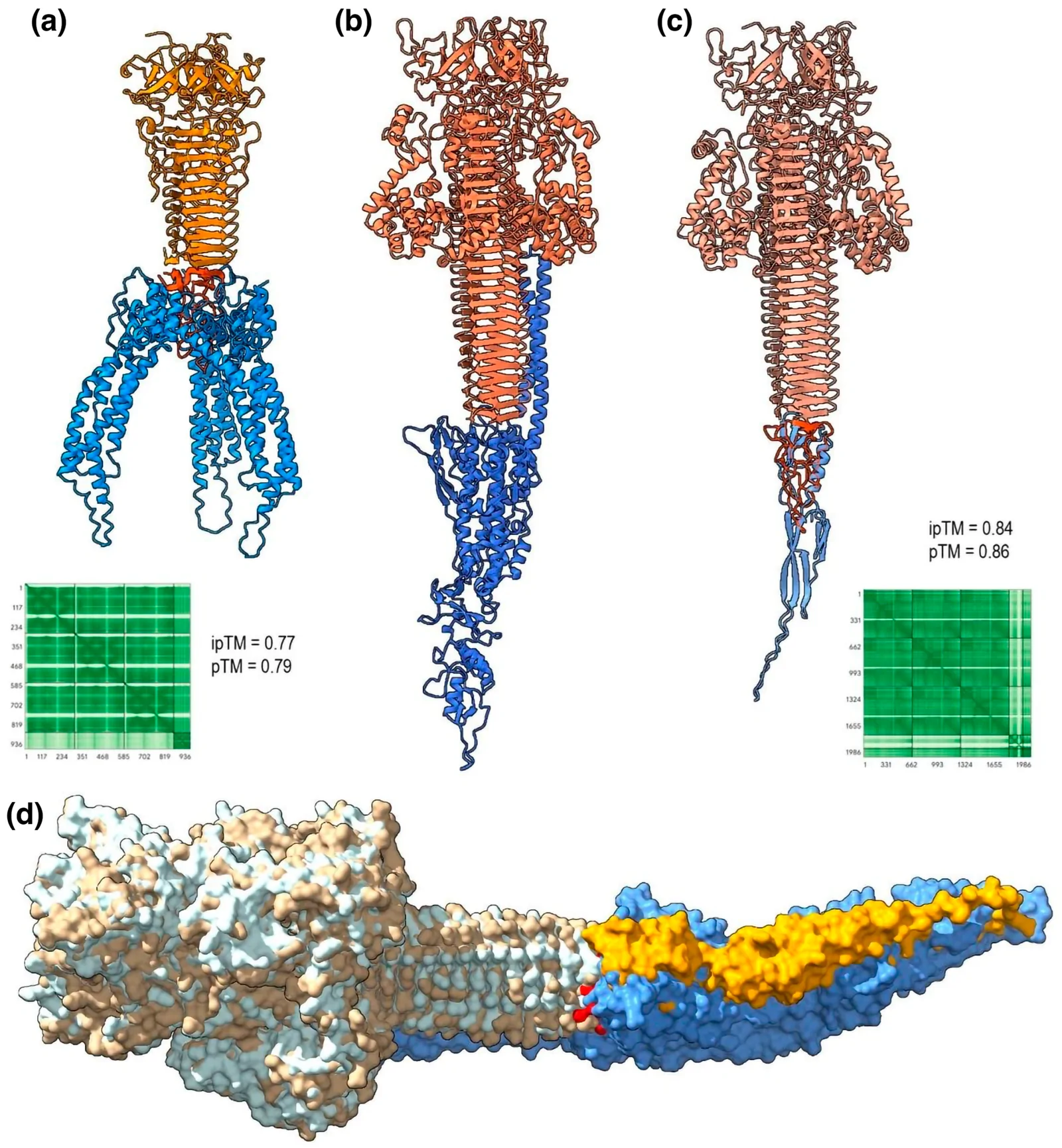
Models of membrane-piercing apparatuses. (**a**) Model of the cell-puncturing device of phage P1, structures of gp5 UfpC shown according to baseplate reconstruction (PDB ID: 9VZK), complex UpfB and UpfC modelled using AlphaFold3. (**b**) Model of the trimer of the phage Helios cell-puncturing device generated by AlphaFold3. (**c**) Model of the cell-puncturing device of phage T4: gp5 trimer (brown), gp5.4 (red), and gp5.1 (blue) generated by AlphaFold3. (**d**) Superposition of spatial AlphaFold3-generated models of complexes of cell-puncturing devices of phages Helios (light brown and blue) and T4 (gp5 (light blue), gp5.4 (red), and gp5.1 (yellow)). Surfaces are shown.

A recently published reconstruction of the particle of phage P1 [57] revealed that the membrane-piercing apparatus, previously thought to consist of gp5 and the PAAR protein UpfC (analogous to gp5.4 of phage T4), is equipped with a specialised α-helical tripod structure organised by three copies of the α-helical protein UpfB attached to the protein UfpC (Figure 16a). Remarkably, three copies of the protein UpfB are also equipped with a loop-like structure at the distal end. The role of the UpfB tripod in piercing the cell membrane is unclear.

Above, we mentioned that the genome of phage T4 encodes a small protein 5.1, the function of which is unclear. Assuming that the trimer of gp5, gp5.1, and gp5.4 may form a complex, we modelled it using the AlphaFold3 programme (Figure 16c). We obtained a model where gp5.1 forms a complex with gp5.4, adjacent to one of the lateral faces of the PAAR pyramid. Although Cryo-EM reconstruction of the particle of phage T4 has been performed more than once [49,55,58], and the structure of the gp5/gp5.4 complex was revealed in the reconstructed model, no density for gp5.1 has been found to date. We speculate that the peripheral and asymmetrical location of gp5.1 makes this element invisible in the reconstruction method used.

The superposition of the models of phage Helios gp202/gp203 and phage T4 gp5/gp5.4/gp5.1 complexes (Figure 16d) demonstrates that both form a long conical structure with a loop on the tip. It has been shown that the presence of gp5.4 in the particle is not absolutely necessary for the infection of phage T4 [56]; it is nevertheless essential for bacteriophage fitness, and gp5.4 specifically interacts with lipopolysaccharides. It is worth noting that phage particles lacking gp5.4 also lacked gp5.1, and the individual effect of gp5.1 on phage fitness has not yet been studied. On the other hand, the presence of a specific loop-like structure at the very end of the membrane-piercing device may play a role in interactions with membrane components.

## 4. Discussion

In this study, we isolated and characterised five novel lytic bacteriophages infecting *Klebsiella grimontii*, a recently described species of the *K. oxytoca* complex that has drawn attention as a reservoir of antimicrobial resistance genes and as an emerging opportunistic pathogen. Apart from a single recent study [59], no phages infecting *K. grimontii* have been studied in structural detail. However, the phages described here are taxonomically distinct from those reported previously and represent three different families, underscoring the diversity of phages capable of infecting this bacterial species.

Five phages studied belong to three distinct genera: *Przondovirus* (Silvester), *Sugarlandvirus* (Lyra, Lyris, Mirion), and *Slopekvirus* (Helios), representing three different families (*Autographiviridae*, *Demerecviridae*, and *Straboviridae*). This taxonomic spread is noteworthy, as it demonstrates that *K. grimontii* is susceptible to phages from multiple phylogenetic lineages, a feature that may be advantageous for the development of phage cocktails.

The biological parameters of the phages were generally similar. Adsorption was fast for all phages and was followed by a short latent period, ranging from 15 to 20 min. One notable feature was the translucent halo around Silvester plaques, which we attributed to the activity of its depolymerase against the capsular polysaccharide of strain K15g. This depolymerase activity was characterised in detail in our previous work [13]. For the other phages, the absence of a halo suggests that the phage particles are devoid of depolymerases and correspondingly recognise a different receptor, possibly a protein on the outer membrane, although this was not investigated in the present study.

The secondary peak observed in the one-step growth curves can be explained by the low multiplicity of infection used in the experiment (MOI = 0.01). Under these conditions, only a small fraction of the host cell population is infected during the initial 5 min adsorption step, leaving the majority of cells uninfected. Following the first lytic cycle, the progeny phages released from the initially infected cells can infect the remaining susceptible cells, producing a second burst after a further latent period.

The biological differences observed between the phages correlate with their structural features. Phage Silvester, equipped with a depolymerase complex that degrades the capsular polysaccharide, achieves more rapid adsorption and a higher burst size. In contrast, the *Sugarlandvirus* phages, which lack depolymerases, adsorb more slowly.

Structural modelling of the receptor-binding proteins provided insight into the diversity of adsorption mechanisms among the phages. Phage Silvester possesses a complex of two depolymerases, where depolymerase I was previously shown to cleave the capsular polysaccharide of strain K15g [13]. The substrate of depolymerase II remains unidentified within our collection of *Klebsiella* strains.

The *Sugarlandvirus* phages Lyra, Lyris, and Mirion share substantial similarity with phage T5 in their structural organisation. The Pb4-Pb5 complex, which forms the distal tip of the tail fibre, is structurally analogous to that of T5, suggesting that Pb5 recognises a proteinaceous receptor on the outer membrane of *K. grimontii*. This inference is supported by the absence of a halo around the plaques of these phages, which would be expected if depolymerase activity were present. If confirmed experimentally, a protein receptor would make these phages less susceptible to capsule variation, a common resistance mechanism in *Klebsiella*.

Phage Helios, a member of the family *Straboviridae*, possesses a contractile tail and a cell-puncturing device structurally similar to that of phage T4 and related phages. We assumed that a complex gp5/gp5.1/gp5.4 of phage T4 exists, and the gp202/gp203 complex that we modelled appears to serve the same function, forming a sharp tip that penetrates the cell’s outer membrane. Thus, we speculate that gp203 is a structural analogue of the putative complex gp5.1/gp5.4 of phage T4.

All five phages were strictly specific to *K. grimontii* and showed no activity against any of the tested *K. pneumoniae* or *K. oxytoca* strains (Supplementary Table S1). Despite this narrow host range, the phages were active against clinical isolates of *K. grimontii*, confirming their relevance. The diversity of receptor targets among the phages minimises the risk of cross-resistance and makes them suitable for a combination cocktail. Overall, this study broadens the panel of thoroughly characterised phages available for therapeutic use.

All five phages lacked genes associated with lysogeny, antibiotic resistance, or virulence factors, consistent with their strictly lytic nature. This is an essential prerequisite for therapeutic applications, as lysogenic phages may contribute to horizontal gene transfer or alter host phenotypes through the introduction of prophage-encoded genes. The absence of such genes in our phages, combined with their specificity for *K. grimontii*, supports their further evaluation as candidates for therapeutic applications.

## 5. Conclusions

Five novel strictly lytic bacteriophages active against *K. grimontii* were isolated and characterised. They belong to three genera and exhibit diverse adsorption apparatus architectures. All phages lack genes associated with lysogeny, toxins, or antibiotic resistance, supporting their potential for therapeutic applications.

## Figures and Tables

**Table 1:** Resistance determinants according to ResFinder.

Resistance Gene	Identity	Predicted Phenotype	PMID	Accession No.	Notes
*bla_OXY-_*_6-4_	100%	amoxicillin, amoxicillin + clavulanic acid, ampicillin, ampicillin + clavulanic acid, cefotaxime, cefoxitin, ceftazidime, piperacillin, piperacillin + tazobactam, ticarcillin, ticarcillin + clavulanic acid.	16048960	AJ871877	Chromosomal class A β-lactamase
*OqxB*	91.02%	ciprofloxacin, nalidixic acid, trimethoprim, chloramphenicol.	18440636	EU370913	OqxB efflux pump

**Table 2:** Bacteriophages lysing K15g.

No.	Name	Taxonomy	Isolation Host	Morphology	Source
1	Silvester	*Przondovirus*	*K. grimontii* K15g	Plaques 1.0–1.2 mm, plaques + halo 1.7–2.0 mm,	Wastewater Mosvodokanal
2	Lyra	*Sugarlandvirus*		plaques 1.0–1.3 mm,	Wastewater Mosvodokanal
3	Lyris	*Sugarlandvirus*		plaques 1.0–1.3 mm,	Wastewater Kurianovo
4	Mirion	*Sugarlandvirus*		plaques 0.9–1.0 mm,	Wastewater Domodedovo
5	Helios	*Slopekvirus*		plaques 1.0–1.2 mm,	Wastewater Mosvodokanal

**Table 3:** Comparative table of the structural proteome of phages T5, Lyra, Lyris, and Mirion.

Phage T5 *	Phage Lyra	Phage Lyris	Phage Mirion
TTP (pb6)	Gp165 (YED27480)	Gp171 (YEI42038)	Gp173 (YEI42223)
P140	Gp164 (YED27479)	Gp170 (YEI42037)	Gp172 (YEI42222)
STF	Gp155 (YED27470)	Gp160 (YEI42027)	Gp163 (YEI42213)
P132	Gp156 (YED27471)	Gp162 (YEI42029)	Gp164 (YEI42214)
DTP (pb9)	Gp159 (YED27474)	Gp165 (YEI42032)	Gp167 (YEI42217)
BPH (pb3)	Gp158 (YED27473)	Gp164 (YEI42031)	Gp166 (YEI42216)
Pb4	Gp157 (YED27472)	Gp163 (YEI42030)	Gp165 (YEI42215)
RBP (pb5)	Gp176 (YED27491)	Gp182 (YEI42049)	Gp184 (YEI42234)

* The names of the structural proteins of the T5 phage are given in accordance with the accepted nomenclature.

## Data Availability

The genome sequences of the bacterial strain *K. grimontii* K15g and the five bacteriophages described in this study have been deposited in the GenBank database under the following accession numbers: NZ_JBAMIA000000000.1 (strain K15g), PV506378 (phage Silvester), PZ305518 (phage Lyra), PZ319654.1 (phage Lyris), PZ319655 (phage Mirion), and PZ273796 (phage Helios).

## Supplementary Materials

The following supporting information can be downloaded at: https://www.mdpi.com/article/10.3390/v18090942/s1, Table S1. Host range of phages Helios, Silvester, Mirion, Lyris, and Lyra against *Klebsiella grimontii*, *K. pneumoniae*, and *K. oxytoca* isolates. Figure S1. Pectolytic activity of K15g on crystal violet pectate (CVP) medium. Figure S2. Fitted adsorption curves of phages Silvester, Mirion, Lyris /Lyra, and Helios. Figure S3. Circular genome map of the phage Silvester generated by Pharokka v1.9.0 (using pyCirclize). Figure S4. Circular genome maps of phages (a) Lyra, (b) Lyris, and (c) Mirion generated by Pharokka v1.9.0 (using pyCirclize). Figure S5. Alignments of genome annotations of phages Lyra, Lyris, and Mirion generated with Clinker in CAGECAT (DOI: 10.1101/2020.11.08.370650). Figure S6. Circular genome map of the Helios phage generated by Pharokka v1.9.0 (using pyCirclize).

## References

[1] Yang J., Long H., Hu Y., Feng Y., McNally A., Zong Z. (2022). *Klebsiella oxytoca* Complex: Update on Taxonomy, Antimicrobial Resistance, and Virulence. Clin. Microbiol. Rev..

[2] Cuénod A., Wüthrich D., Seth-Smith H.M.B., Ott C., Gehringer C., Foucault F., Mouchet R., Kassim A., Revathi G., Vogt D.R. (2021). Whole-Genome Sequence-Informed MALDI-TOF MS Diagnostics Reveal Importance of *Klebsiella oxytoca* Group in Invasive Infections: A Retrospective Clinical Study. Genome Med..

[3] Passet V., Brisse S. (2018). Description of *Klebsiella grimontii* sp. Nov.. Int. J. Syst. Evol. Microbiol..

[4] Liu L., Feng Y., Hu Y., Kang M., Xie Y., Zong Z. (2018). *Klebsiella grimontii*, a New Species Acquired Carbapenem Resistance. Front. Microbiol..

[5] Campos-Madueno E.I., Moser A.I., Risch M., Bodmer T., Endimiani A. (2021). Exploring the Global Spread of *Klebsiella grimontii* Isolates Possessing blaVIM-1 and Mcr-9. Antimicrob. Agents Chemother..

[6] Campos-Madueno E.I., Mauri C., Meroni E., Paseiro P.P., Consonni A., Luzzaro F., Endimiani A. (2022). Simultaneous Gut Colonization by *Klebsiella grimontii* and *Escherichia coli* Co-Possessing the blaKPC-3-Carrying pQil Plasmid. Eur. J. Clin. Microbiol. Infect. Dis..

[7] Aranaga C., Pantoja L.D., Martínez E.A., Falco A. (2022). Phage Therapy in the Era of Multidrug Resistance in Bacteria: A Systematic Review. Int. J. Mol. Sci..

[8] Pirnay J.-P., Djebara S., Steurs G., Griselain J., Cochez C., De Soir S., Glonti T., Spiessens A., Vanden Berghe E., Green S. (2024). Personalized Bacteriophage Therapy Outcomes for 100 Consecutive Cases: A Multicentre, Multinational, Retrospective Observational Study. Nat. Microbiol..

[9] Bris J.L., Chen N., Supandy A., Rendueles O., Tyne D.V. (2025). Phage Therapy for *Klebsiella pneumoniae*: Understanding Bacteria–Phage Interactions for Therapeutic Innovations. PLoS Pathog..

[10] Yang X., Wu N., Jiang X., Cheng M., Hu L., Li N., Fang Y., Yu X., Ji J., Ding X. (2026). Combined Bacteriophage and Antibiotic Therapy for Refractory Peritoneal Dialysis-Related Peritonitis Caused by *Klebsiella pneumoniae*. Nat. Commun..

[11] Li P., Zhang Y., Yan F., Zhou X. (2021). Characteristics of a Bacteriophage, vB_Kox_ZX8, Isolated From Clinical *Klebsiella oxytoca* and Its Therapeutic Effect on Mice Bacteremia. Front. Microbiol..

[12] Harper D.R. (2018). Criteria for Selecting Suitable Infectious Diseases for Phage Therapy. Viruses.

[13] Lukianova A.A., Tokmakova A.D., Kasimova A.A., Shelenkov A.A., Mikhailova Y.V., Putilov I.A., Knirel Y.A., Miroshnikov K.A., Shneider M.M. (2026). Structure of *Klebsiella grimontii* K15g Capsular Polysaccharide and Products of Its Cleavage by Phage Silvester Depolymerase. Biochem. Mosc..

[14] Sayers E.W., Beck J., Bolton E.E., Brister J.R., Chan J., Connor R., Feldgarden M., Fine A.M., Funk K., Hoffman J. (2025). Database Resources of the National Center for Biotechnology Information in 2025. Nucleic Acids Res..

[15] Freese H.M., Meier-Kolthoff J.P., Sardà Carbasse J., Afolayan A.O., Göker M. (2026). TYGS and LPSN in 2025: A Global Core Biodata Resource for Genome-Based Classification and Nomenclature of Prokaryotes within DSMZ Digital Diversity. Nucleic Acids Res..

[16] Kim J., Na S.-I., Kim D., Chun J. (2021). UBCG2: Up-to-Date Bacterial Core Genes and Pipeline for Phylogenomic Analysis. J. Microbiol..

[17] Katoh K., Standley D.M. (2013). MAFFT Multiple Sequence Alignment Software Version 7: Improvements in Performance and Usability. Mol. Biol. Evol..

[18] Borowiec M.L. (2016). AMAS: A Fast Tool for Alignment Manipulation and Computing of Summary Statistics. PeerJ.

[19] Wong T.K.F., Ly-Trong N., Ren H., Demotte P., Baños H., Roger A.J., Susko E., Bielow C., De Maio N., Goldman N. (2026). IQ-TREE 3: Phylogenomic Inference Software Using Complex Evolutionary Models. Mol. Biol. Evol..

[20] Letunic I., Bork P. (2024). Interactive Tree of Life (iTOL) v6: Recent Updates to the Phylogenetic Tree Display and Annotation Tool. Nucleic Acids Res..

[21] Ackermann H.-W., Clokie M.R.J., Kropinski A.M. (2009). Basic Phage Electron Microscopy. Bacteriophages: Methods and Protocols.

[22] Winnepenninckx B., Backeljau T., De Wachter R. (1993). Extraction of High Molecular Weight DNA from Molluscs. Trends Genet. Amst..

[23] Bankevich A., Nurk S., Antipov D., Gurevich A.A., Dvorkin M., Kulikov A.S., Lesin V.M., Nikolenko S.I., Pham S., Prjibelski A.D. (2012). SPAdes: A New Genome Assembly Algorithm and Its Applications to Single-Cell Sequencing. J. Comput. Biol..

[24] Nayfach S., Camargo A.P., Schulz F., Eloe-Fadrosh E., Roux S., Kyrpides N.C. (2021). CheckV Assesses the Quality and Completeness of Metagenome-Assembled Viral Genomes. Nat. Biotechnol..

[25] Bouras G., Nepal R., Houtak G., Psaltis A.J., Wormald P.-J., Vreugde S. (2023). Pharokka: A Fast Scalable Bacteriophage Annotation Tool. Bioinformatics.

[26] Söding J., Biegert A., Lupas A.N. (2005). The HHpred Interactive Server for Protein Homology Detection and Structure Prediction. Nucleic Acids Res..

[27] Moraru C., Varsani A., Kropinski A.M. (2020). VIRIDIC—A Novel Tool to Calculate the Intergenomic Similarities of Prokaryote-Infecting Viruses. Viruses.

[28] Turner D., Kropinski A.M., Adriaenssens E.M. (2021). A Roadmap for Genome-Based Phage Taxonomy. Viruses.

[29] Meier-Kolthoff J.P., Göker M. (2017). VICTOR: Genome-Based Phylogeny and Classification of Prokaryotic Viruses. Bioinformatics.

[30] Abramson J., Adler J., Dunger J., Evans R., Green T., Pritzel A., Ronneberger O., Willmore L., Ballard A.J., Bambrick J. (2024). Accurate Structure Prediction of Biomolecular Interactions with AlphaFold 3. Nature.

[31] Fevre C., Jbel M., Passet V., Weill F.-X., Grimont P.A.D., Brisse S. (2005). Six Groups of the OXY β-Lactamase Evolved over Millions of Years in *Klebsiella oxytoca*. Antimicrob. Agents Chemother..

[32] Bharatham N., Bhowmik P., Aoki M., Okada U., Sharma S., Yamashita E., Shanbhag A.P., Rajagopal S., Thomas T., Sarma M. (2021). Structure and Function Relationship of OqxB Efflux Pump from *Klebsiella pneumoniae*. Nat. Commun..

[33] Kim H.B., Wang M., Park C.H., Kim E.-C., Jacoby G.A., Hooper D.C. (2009). oqxAB Encoding a Multidrug Efflux Pump in Human Clinical Isolates of Enterobacteriaceae. Antimicrob. Agents Chemother..

[34] Albarri O., AlMatar M., Öcal M.M., Köksal F. (2022). Overexpression of Efflux Pumps AcrAB and OqxAB Contributes to Ciprofloxacin Resistance in Clinical Isolates of *K. pneumonia*. Curr. Protein Pept. Sci..

[35] Pfeifer Y., Cullik A., Witte W. (2010). Resistance to Cephalosporins and Carbapenems in Gram-Negative Bacterial Pathogens. Int. J. Med. Microbiol..

[36] Chen W., Xiao H., Wang X., Song S., Han Z., Li X., Yang F., Wang L., Song J., Liu H. (2020). Structural Changes of a Bacteriophage upon DNA Packaging and Maturation. Protein Cell.

[37] Chao K.L., Shang X., Greenfield J., Linden S.B., Alreja A.B., Nelson D.C., Herzberg O. (2022). Structure of *Escherichia coli* O157:H7 Bacteriophage CBA120 Tailspike Protein 4 Baseplate Anchor and Tailspike Assembly Domains (TSP4-N). Sci. Rep..

[38] Plattner M., Shneider M.M., Arbatsky N.P., Shashkov A.S., Chizhov A.O., Nazarov S., Prokhorov N.S., Taylor N.M.I., Buth S.A., Gambino M. (2019). Structure and Function of the Branched Receptor-Binding Complex of Bacteriophage CBA120. J. Mol. Biol..

[39] Lukianova A., Shneider M., Tokmakova A., Landyshev N., Kasimova A., Kolganova A., Mikhaylova Y., Shelenkov A., Dmitrenok A., Chizhov A. (2026). Novel Autographivirales Phages Kiwi, Nika and Pie Targeting *Klebsiella pneumoniae* K14: Isolation, Characterisation, Genome Analysis and Study of the Bacterial Polysaccharide Degradation. Arch. Virol..

[40] Otwinowska A., Olejniczak S., Latka A., Pozniak M., Majkowska-Skrobek G., Maciejewska B., Koszucki J., Panicker V., Jabłońska S., Hulsens M. (2025). DepoCatalog: Mapping the Diversity of 105 Recombinant *Klebsiella* Phage Depolymerases Across Sequence, Structure, and Substrate Specificity. bioRxiv.

[41] Concha-Eloko R., Barberán-Martínez P., Sanjuán R., Domingo-Calap P. (2023). Broad-Range Capsule-Dependent Lytic Sugarlandvirus against *Klebsiella* sp.. Microbiol. Spectr..

[42] Degroux S., Effantin G., Linares R., Schoehn G., Breyton C. (2023). Deciphering Bacteriophage T5 Host Recognition Mechanism and Infection Trigger. J. Virol..

[43] Linares R., Arnaud C.-A., Effantin G., Darnault C., Epalle N.H., Boeri Erba E., Schoehn G., Breyton C. (2023). Structural Basis of Bacteriophage T5 Infection Trigger and *E. Coli* Cell Wall Perforation. Sci. Adv..

[44] Maffei E., Shaidullina A., Burkolter M., Heyer Y., Estermann F., Druelle V., Sauer P., Willi L., Michaelis S., Hilbi H. (2021). Systematic Exploration of *Escherichia coli* Phage–Host Interactions with the BASEL Phage Collection. PLoS Biol..

[45] Buth S.A., Shneider M.M., Scholl D., Leiman P.G. (2018). Structure and Analysis of R1 and R2 Pyocin Receptor-Binding Fibers. Viruses.

[46] North O.I., Sakai K., Yamashita E., Nakagawa A., Iwazaki T., Büttner C.R., Takeda S., Davidson A.R. (2019). Phage Tail Fibre Assembly Proteins Employ a Modular Structure to Drive the Correct Folding of Diverse Fibres. Nat. Microbiol..

[47] Klein-Sousa V., Roa-Eguiara A., Kielkopf C.S., Sofos N., Taylor N.M.I. (2025). RBPseg: Toward a Complete Phage Tail Fiber Structure Atlas. Sci. Adv..

[48] Garcia-Doval C., Castón J.R., Luque D., Granell M., Otero J.M., Llamas-Saiz A.L., Renouard M., Boulanger P., van Raaij M.J. (2015). Structure of the Receptor-Binding Carboxy-Terminal Domain of the Bacteriophage T5 L-Shaped Tail Fibre with and without Its Intra-Molecular Chaperone. Viruses.

[49] Roa-Eguiara A., Marín-Arraiza L., Klein-Sousa V., Santiveri M., Rutbeek N.R., Piel D., Pape T., Sofos N., Hendriks I.A., Nielsen M.L. (2026). Genome Delivery of a Contractile Tailed Phage and Its Superinfection Exclusion Mechanism. bioRxiv.

[50] Peters D.L., Davis C.M., Harris G., Zhou H., Rather P.N., Hrapovic S., Lam E., Dennis J.J., Chen W. (2023). Characterization of Virulent T4-Like *Acinetobacter baumannii* Bacteriophages DLP1 and DLP2. Viruses.

[51] Dunne M., Denyes J.M., Arndt H., Loessner M.J., Leiman P.G., Klumpp J. (2018). *Salmonella* Phage S16 Tail Fiber Adhesin Features a Rare Polyglycine Rich Domain for Host Recognition. Structure.

[52] Bartual S.G., Otero J.M., Garcia-Doval C., Llamas-Saiz A.L., Kahn R., Fox G.C., van Raaij M.J. (2010). Structure of the Bacteriophage T4 Long Tail Fiber Receptor-Binding Tip. Proc. Natl. Acad. Sci. USA.

[53] McCalla A.J., Walker F.C., Bisaro F., Rodriguez-Anavitate M., Johannesman A., Di Venanzio G., Feldman M.F., LeRoux M. (2025). *Acinetobacter* Phages Use Distinct Strategies to Breach the Capsule Barrier. PLoS Pathog..

[54] Islam M.Z., Fokine A., Mahalingam M., Zhang Z., Garcia-Doval C., van Raaij M.J., Rossmann M.G., Rao V.B. (2019). Molecular Anatomy of the Receptor Binding Module of a Bacteriophage Long Tail Fiber. PLoS Pathog..

[55] Taylor N.M.I., Prokhorov N.S., Guerrero-Ferreira R.C., Shneider M.M., Browning C., Goldie K.N., Stahlberg H., Leiman P.G. (2016). Structure of the T4 Baseplate and Its Function in Triggering Sheath Contraction. Nature.

[56] Mattenberger Y., Knyazhanskaya E.S., Shneider M.M., Buth S.A., Nazarov S., Robins W.P., Leiman P.G., Belin D. (2025). The Spike Tip Protein of Bacteriophage T4. bioRxiv.

[57] Zheng J., Chen Y., Chen S., Zhou J., Xiao H., Yang F., Liu H. (2026). Structure of Defense against Restriction Proteins DarA and Hdf in Phage P1 Reveals a New Molecular Mechanism during Phage Assembly, Infection and DNA Ejection. PLoS Pathog..

[58] Fokine A., Zhu J., Klose T., Vago F., Arnaud C., Wang Z., Khare B., Rossmann M.G., Chen Z., Sun L. (2025). Structure of the Bacteriophage T4 Portal-Neck-Tail Complex. PDB Protein Data Bank.

[59] de Scally S.Z., Blake C., Barr J.J., McDonald M.J. (2026). Isolation and Characterisation of Virulent Bacteriophage Targeting Canola Rhizosphere Bacteria *Klebsiella grimontii* and *Bacillus* sp.. Virology.

