# Supplementary material for: Isolation and Genomic Characterisation of Five Novel Lytic Bacteriophages Infecting the Emerging Pathogen Klebsiella grimontii
Source: Viruses. 2026 Aug 28;18(9):942. doi: 10.3390/v18090942 (PMC13611949; doi:10.3390/v18090942)
Supplement: Supplementary file 1 [file viruses-18-00942-s001.zip › Figure S4 (map Lyra_Lyris_Mirion).pdf]

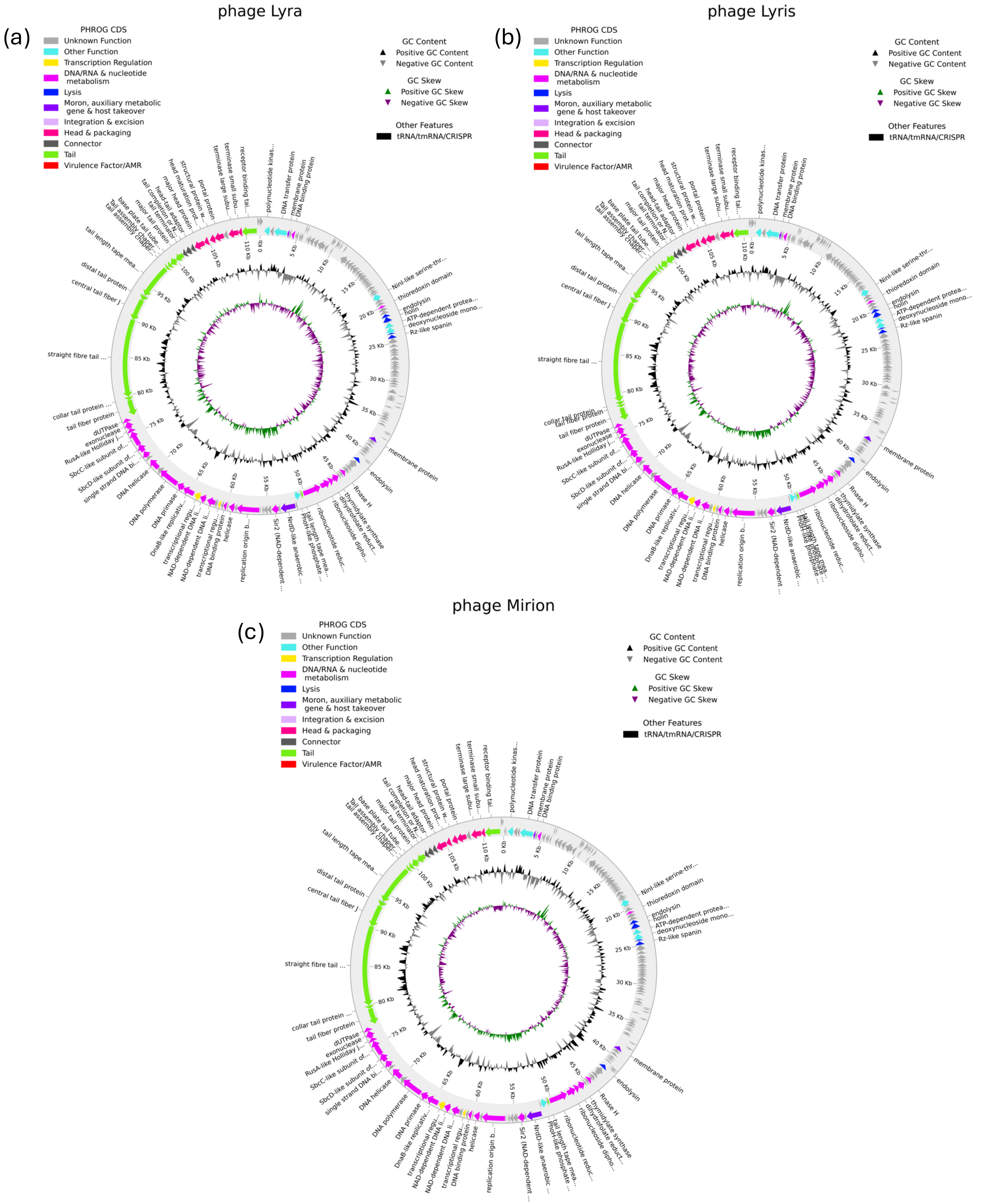
phage Lyra
(a)
PHROG CDS
Unknown Function
Other Function
Transcription Regulation
DNA/RNA & nucleotide metabolism
Lysis
Moron, auxiliary metabolic gene & host takeover
Integration & excision
Head & packaging
Connector
Tail
Virulence Factor/AMR
GC Content
Positive GC Content
Negative GC Content
GC Skew
Positive GC Skew
Negative GC Skew
Other Features
tRNA/tmRNA/CRISPR
polynucleotide kinas...
DNA transfer protein
membrane protein
DNA binding protein
NinI-like serine-thr...
thioredoxin domain
endolysin
holin
ATP-dependent protea...
deoxynucleoside mono...
Rz-like spanin
membrane protein
endolysin
Rnase H
thymidylate synthase
dihydrofolate reduct...
ribonucleoside dipho...
ribonucleotide reduc...
tail length tape mea...
PhoH-like phosphate ...
NrdD-like anaerobic ...
Sir2 (NAD-dependent ...
replication origin b...
helicase
DNA binding protein
transcriptional regu...
NAD-dependent DNA li...
NAD-dependent DNA li...
transcriptional regu...
DnaB-like replicativ...
DNA primase
DNA polymerase
DNA helicase
single strand DNA bi...
SbcD-like subunit of...
SbcC-like subunit of...
RusA-like Holliday j...
exonuclease
dUTPase
tail fiber protein
collar tail protein ...
straight fibre tail ...
central tail fiber J
distal tail protein
tail length tape mea...
tail assembly chaper...
Tail assembly chaper...
base plate tail tube...
major tail protein
tail completion or N...
tail terminator
head-tail adaptor
major head protein
head maturation prot...
structural protein w...
portal protein
terminase large subu...
terminase small subu...
receptor binding tai...
phage Lyris
(b)
phage Mirion
(c)
