# Supplementary material for: Isolation and Genomic Characterisation of Five Novel Lytic Bacteriophages Infecting the Emerging Pathogen Klebsiella grimontii
Source: Viruses. 2026 Aug 28;18(9):942. doi: 10.3390/v18090942 (PMC13611949; doi:10.3390/v18090942)
Supplement: Supplementary file 1 [file viruses-18-00942-s001.zip › Table S1.pdf]

## Supplementary Table S1. Host range of phages Helios, Silvester, Mirion, Lyris and Lyra against *Klebsiella grimontii, K. pneumoniae*, and *K. oxytoca* isolates.

***S***: *clear lysis;* ***R***: *resistant (no lysis).*

| Strain ID | Species | Serotype / Source | Helios | Silvester | Mirion | Lyris | Lyra | Notes |
|---|---|---|---|---|---|---|---|---|
| Group 1: *K. grimontii* strains | | | | | | | | |
| K15g | *K. grimontii* | Decaying *Iris germanica* | S | S | S | S | S | Structure of capsule polysaccharide described at doi:10.1134/S0006297925601935 |
| 1621 | *K. grimontii* | Feces, diarrhea | S | R | S | S | S | |
| 3770 | *K. grimontii* | Urine | R | R | S | S | S | Turbid lysis at high titer only (Mirion, Lyris) |
| 1801 | *K. grimontii* | Diabetic foot ulcer, remission | R | R | R | R | R | Resistant |
| 1836 | *K. grimontii* | Diabetic foot ulcer, remission | R | R | R | R | R | Resistant |
| 2641 | *K. grimontii* | Feces, diarrhea | R | R | R | R | R | Resistant |
| 3113 | *K. grimontii* | Feces, diarrhea | R | R | R | R | R | Resistant |
| 3768 | *K. grimontii* | Urine | R | R | R | R | R | Resistant |
| Group 2: Other *Klebsiella* spp. | | | | | | | | |
| 62867 | *K. pneumoniae* | K1 | R | R | R | R | R | - |
| 77245 | *K. pneumoniae* | K2 | R | R | R | R | R | - |
| 69402 | *K. pneumoniae* | K12 | R | R | R | R | R | - |

| | | | | | | | | |
|---|---|---|---|---|---|---|---|---|
| KL12 | *K. pneumoniae* | K12 | R | R | R | R | R | - |
| KL13 | *K. pneumoniae* | K13 | R | R | R | R | R | - |
| kot L | *K. pneumoniae* | K14 | R | R | R | R | R | - |
| LisaK1 | *K. pneumoniae* | K14 | R | R | R | R | R | - |
| LisaK2 | *K. pneumoniae* | K14 | R | R | R | R | R | - |
| 88166 | *K. pneumoniae* | K15 | R | R | R | R | R | - |
| KphM | *K. pneumoniae* | K16 | R | R | R | R | R | - |
| 77840 | *K. pneumoniae* | K16 | R | R | R | R | R | - |
| 74610 | *K. pneumoniae* | K17 | R | R | R | R | R | - |
| 80384 | *K. pneumoniae* | K20 | R | R | R | R | R | - |
| Kph1 | *K. pneumoniae* | K20 | R | R | R | R | R | - |
| K5 | *K. pneumoniae* | K21 | R | R | R | R | R | - |
| Kph5 | *K. pneumoniae* | K23 | R | R | R | R | R | - |
| 78315 | *K. pneumoniae* | K24 | R | R | R | R | R | - |
| 77680 | *K. pneumoniae* | K25 | R | R | R | R | R | - |
| 77487 | *K. pneumoniae* | K39 | R | R | R | R | R | - |
| 1620 | *K. oxytoca* | K55 | R | R | R | R | R | - |
| 81841 | *K. pneumoniae* | K62 | R | R | R | R | R | - |
| 77864 | *K. pneumoniae* | K64 | R | R | R | R | R | - |
| Kph13 | *K. pneumoniae* | K107 | R | R | R | R | R | - |
| 1333 | *K. pneumoniae* | K108 | R | R | R | R | R | - |
| 1226 | *K. pneumoniae* | K112 | R | R | R | R | R | - |
| 19.01 | *K. pneumoniae* | K114 | R | R | R | R | R | - |
